# Quinoa genome assembly employing genomic variation for guided scaffolding

**DOI:** 10.1007/s00122-021-03915-x

**Published:** 2021-08-07

**Authors:** Alexandrina Bodrug-Schepers, Nancy Stralis-Pavese, Hermann Buerstmayr, Juliane C. Dohm, Heinz Himmelbauer

**Affiliations:** 1 Institute of Computational Biology, Department of Biotechnology, Universität für Bodenkultur, Vienna, Austria; 2 Institute of Biotechnology in Plant Production, Department of Agrobiotechnology and Department of Crop Sciences, Universität für Bodenkultur, Tulln, Austria

## Abstract

**Key message:**

We propose to use the natural variation between individuals of a population for genome assembly scaffolding. In today’s genome projects, multiple accessions get sequenced, leading to variant catalogs. Using such information to improve genome assemblies is attractive both cost-wise as well as scientifically, because the value of an assembly increases with its contiguity. We conclude that haplotype information is a valuable resource to group and order contigs toward the generation of pseudomolecules.

**Abstract:**

Quinoa (*Chenopodium quinoa*) has been under cultivation in Latin America for more than 7500 years. Recently, quinoa has gained increasing attention due to its stress resistance and its nutritional value. We generated a novel quinoa genome assembly for the Bolivian accession CHEN125 using PacBio long-read sequencing data (assembly size 1.32 Gbp, initial N50 size 608 kbp). Next, we re-sequenced 50 quinoa accessions from Peru and Bolivia. This set of accessions differed at 4.4 million single-nucleotide variant (SNV) positions compared to CHEN125 (1.4 million SNV positions on average per accession). We show how to exploit variation in accessions that are distantly related to establish a genome-wide ordered set of contigs for guided scaffolding of a reference assembly. The method is based on detecting shared haplotypes and their expected continuity throughout the genome (i.e., the effect of linkage disequilibrium), as an extension of what is expected in mapping populations where only a few haplotypes are present. We test the approach using *Arabidopsis thaliana* data from different populations. After applying the method on our CHEN125 quinoa assembly we validated the results with mate-pairs, genetic markers, and another quinoa assembly originating from a Chilean cultivar. We show consistency between these information sources and the haplotype-based relations as determined by us and obtain an improved assembly with an N50 size of 1079 kbp and ordered contig groups of up to 39.7 Mbp. We conclude that haplotype information in distantly related individuals of the same species is a valuable resource to group and order contigs according to their adjacency in the genome toward the generation of pseudomolecules.

**Supplementary Information:**

The online version contains supplementary material available at 10.1007/s00122-021-03915-x.

## Introduction

Quinoa *(Chenopodium quinoa* Willd.) is a crop plant that has been under cultivation in Latin America for more than 7500 years (Jellen et al. 2011; Lack and Fuentes 2011). Today, quinoa is grown in an area ranging from Colombia to Chile, as well as in parts of North America, France and other countries. The nutritious seeds of quinoa are free of gluten, making them an interesting alternative to cereals, especially in the context of celiac disease. The plants are hardy and can be grown on poor soil of high salinity; they are also resistant against drought and temperature fluctuation. The remarkable properties of quinoa have made the Food and Agriculture Organization (FAO) to declare 2013 as the “International Year of Quinoa.” Quinoa has been grouped based on agronomic, morphological and distributional criteria into five ecotypes, including the “Altiplano” ecotype grown at 4000 m of altitude in the high Andes of central South America, a “Valle” ecotype thriving at around 2400 m, the “Yungas” and “Salares” groups, and Chilean “Nivel del mar” accessions (Tapia et al. 1980). The highest variability in quinoa is found in Peru and Bolivia, in agreement with the hypothesis that quinoa domestication first took place in the Andean Altiplano region Ayacucho (Lumbreras et al. 2008; Murphy and Matanguihan 2015). Cultivar variability decreases the further away from this center, with the exception of the Chilean coastal quinoa accessions that carry unique alleles (Bazile et al. 2013; Fuentes et al. 2009). Quinoa is affiliated with the Amaranthaceae family (order Caryophyllales), an economically important taxon that also includes the crops sugar beet and spinach. Previous estimations using flow cytometry showed that quinoa has a haploid genome size of 1.448 Gbp (Palomino et al. 2008) or 1.454 Gbp (Kolano et al. 2012), respectively. Quinoa is an allotetraploid plant with 2*n* = 4*x* = 36 chromosomes (Ward 2000) that resulted from a hybridization of two *Chenopodium* species about five million years ago. The parents that gave rise to quinoa were close enough to hybridize, but different enough to remain distinct within the same nucleus up to now (Schiavinato et al. 2021). Thus, quinoa is a tetraploid species from an evolutionary perspective; however, its haploid chromosome number is established as 18 molecules. Homeologous chromosomes can be clearly distinguished by their sequence as exemplified by quinoa genome assemblies that have sizes similar to the sum of the haploid genome sizes of the presumptive ancestral parental species. Descendants of these ancestral parental species exist today, and we know that the genome of quinoa contains nine chromosomes from subgenome type A (exemplified by extant *C. pallidicaule*, which, however, was recently excluded as direct progenitor due to its unique repeat profiles (Heitkam et al. 2020)) and nine chromosomes from subgenome type B (exemplified by extant species *C. suecium* or *C. ficifolium* (Walsh et al. 2015)).

Quinoa is mainly self-pollinating, resulting in reduced overall heterozygosity of the genome. By C-value analysis, the genome of quinoa has been estimated as 1.45 Gbp in size (Palomino et al. 2008). Comparison between the Salt Overly Sensitive (SOS) 1 loci in its A and B subgenome versions suggested high sequence divergence between the two subgenomes: sequence conservation between A and B subgenomes was restricted to coding regions with 82.5% nucleotide identity on average, while intergenic regions were poorly covered (Maughan et al. 2012). Meanwhile, three quinoa genome assemblies have been published, i.e., one from the Chilean cultivar QQ74 (Jarvis et al. 2017), one from the Bolivian cultivar Real (Zou et al. 2017) and one from the inbred accession Kd (Yasui et al. 2016). Here, we present a novel quinoa genome assembly of the publicly available Altiplano accession CHEN125 originally collected at Lahuachaca, Bolivia, along with re-sequencing data from 50 quinoa accessions of Peruvian and Bolivian origin.

The assembly of plant genomes is complicated by transposable elements and other repetitive sequences. A practical consequence of the presence of repeats in the genome is assembly fragmentation which needs to be overcome by subsequent steps that result in the generation of scaffolds, i.e., ordered and orientated arrays of contigs linked by stretches of undetermined bases of estimated lengths ("gaps"). Even if gaps between contigs mostly composed of repeats remain unassembled, knowledge about the contig order and orientation is extremely valuable information. Scaffolding can be performed by connecting assembled contigs based on different types of sequence-based resources or genome maps. In the context of short-read assemblies as they are generated from Illumina sequencing data, mate-pair data with span sizes up to 10 kbp are most frequently employed for scaffolding. With the advent of long-read sequencing platforms such as those from Pacific Biosciences and Oxford Nanopore Technologies, assemblies typically contain much larger contigs than assemblies computed from short-read datasets. New technologies were adopted to support the ordering of long contigs, e.g., by integration with optical maps, or by the use of linked-read data or Hi-C data. All these approaches provide long-range information so that contigs can be linked together and gap lengths can be estimated.

However, another source of such information is contained within the genome itself when comparing several individuals of a species. In a given region of the genome, different individuals of the same species carry a limited number of versions of the same chromosome, called haplotypes, that can be distinguished by the particular set of alleles, i.e., differences on the nucleotide level that are shared by groups of individuals. This has been explored in plants by using F2 mapping populations, advanced intercrosses, or F1 outbred populations in previous studies (Huang et al. 2009; Dohm et al. 2014; Vlasova et al. 2016; Zhou et al. 2020). In this work, we expand this concept by using randomly chosen individuals instead of progeny of targeted crossing and demonstrate how haplotypes contained in the population can be used for genome assembly scaffolding. In other words, linkage disequilibrium (LD) mapping is used to arrange contigs into scaffolds, exploiting the principle that variants originating from the same ancestral chromosome will remain linked to each other depending on their distance in the genome (Lewontin and Kojima 1960; Flint-Garcia et al. 2003; Nordborg et al. 2005).

Programs like LDMAP (Pengelly and Collins 2019) and LDscaff (Zhao et al. 2020) were developed to address this task based on allele frequencies. In this study, we infer haplotype blocks based on the comparison of variation patterns without taking allele frequencies into account and demonstrate that this approach leads to substantial improvements in the contiguity of the quinoa genome assembly.

We first demonstrate the feasibility of our method using the model plant *Arabidopsis thaliana.* Then, using long molecule sequencing, we assemble the CHEN125 quinoa genome into contigs and scaffold them using haplotype information provided by the comparison of 50 re-sequenced accessions of quinoa.

## Results

### Genome assembly scaffolding guided by haplotype information

**Fig. 1:**
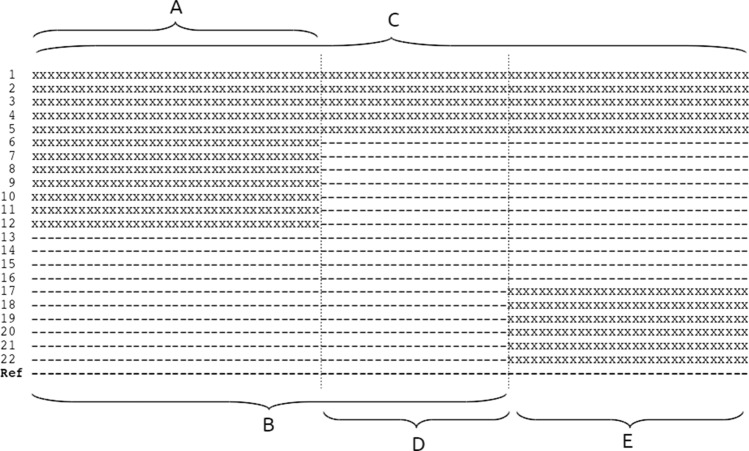
Variants of 22 individuals (1–22) compared to a reference assembly (Ref), only variation positions are shown (schematic). In the genomic interval A, two haplotypes can be distinguished represented by individuals 1–12 and 13–22, respectively. In the genomic interval B, three haplotypes can be distinguished, represented by individuals 1–5, 6–12 and 13–22, respectively. In the whole genomic region (C), four haplotypes can be distinguished, represented by individuals 1–5, 6–12, 13–16 and 17–22, respectively. In total there are three different patterns of variation per genomic position: the first pattern in interval A, the second in interval D, the third in interval E

The scaffolding approach that we propose is based on the presence of shared haplotypes in the genomes of a species. Thus, in principle, it makes use of the effect of linkage disequilibrium (LD) (Lewontin and Kojima 1960). Each individual plant that is compared to a reference genome can show either the reference allele or a non-reference allele at a given genomic position. Since variation is inherited within the species we expect groups of individuals that show non-reference alleles at the same genomic reference positions. Depending on the size of the genomic region analyzed we find a number of such groups, each representing one haplotype in this region (Fig. 1). In a mapping population where the parental haplotypes are distinguished by a known set of variant positions (or any set of markers) one can assign each region of the genomes of the following generation to one of the parental haplotypes. In this study, when looking at individuals that are not directly related, we do not know how many “initial” haplotypes should be considered so that we determine haplotypes simply based on shared variation within a given genomic region. In other words, any small genomic region is expected to show two groups of individuals, the one reference-like and the other one different from the reference. In a larger genomic region, more than two groups may become apparent as more positions are considered. This grouping of individuals based on shared variants results in a variation pattern for each genomic position in the genome. The change from one pattern into another one is expected to happen through longer genomic distances (corresponding to expected distances for meiotic crossing-over) so that sequences of a fragmented genome assembly used as reference may show variation patterns that continue across assembly gaps. In this way, continuous variation patterns (i.e., variant positions that group the individuals in a similar way) may serve as a guidance for ordering and orientating assembled contigs. Genomic positions that do not show any variants or noisy positions need to be removed, and variation patterns need to be summarized in order to reduce the number of patterns to be compared between contigs.

The main steps are: sequencing of a number of individuals, read mapping on the reference assembly, variant calling for each individual compared to the reference, filtering of uninformative or noisy genomic positions, summarizing variation patterns within genomic intervals, and comparison of the summarized variation patterns between contigs.

**Fig. 2:**
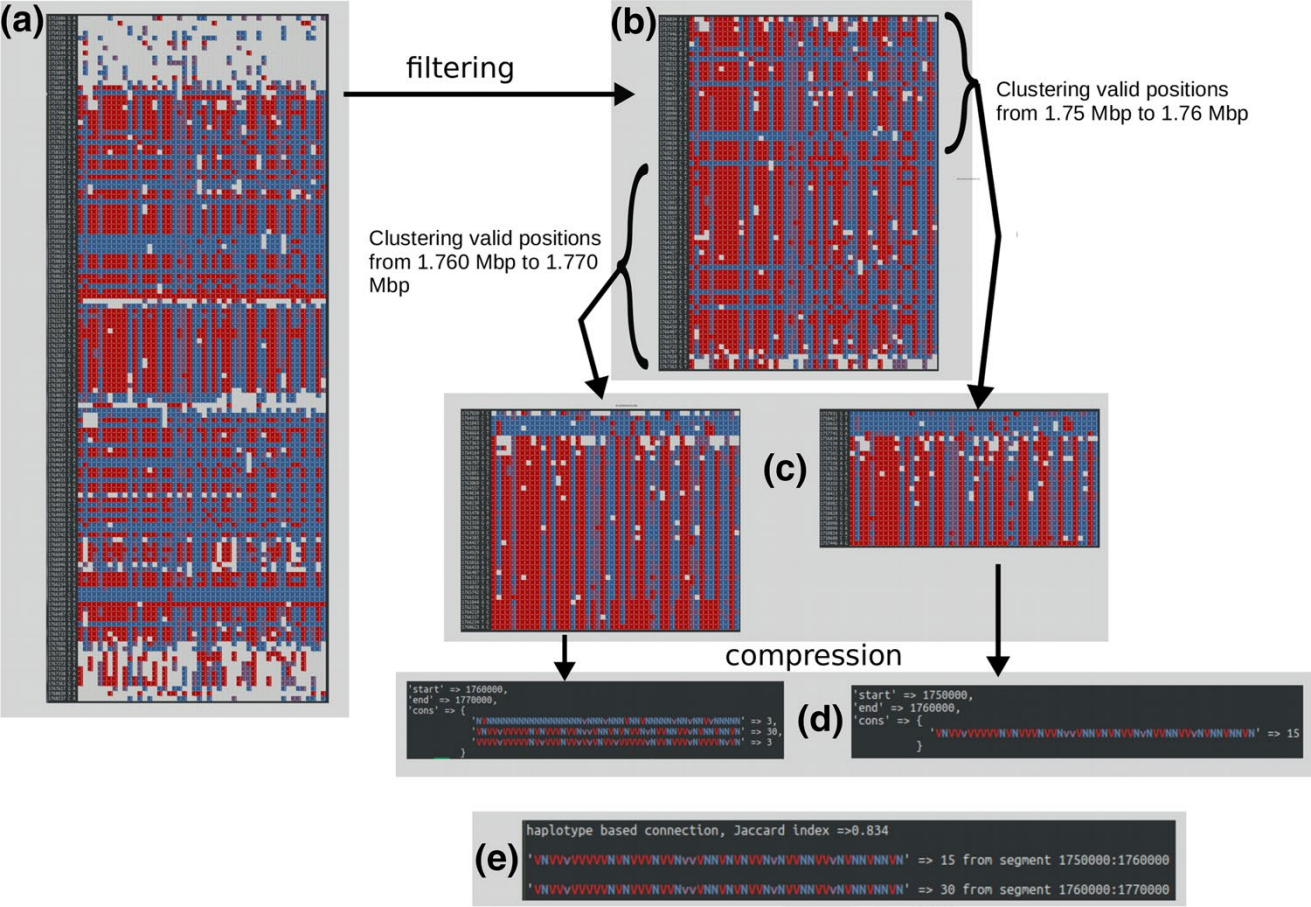
Two genomic segments are clustered and compressed to obtain their variation fingerprints. Variants (red: homozygous, purple: heterozygous), reference alleles (blue), and missing information (gray) are indicated for each of 50 quinoa accessions (columns) along a genomic region of 20 kbp in the Qpac assembly (tig00006496, 1.75 Mbp to 1.77 Mbp). Only positions that carry alternative alleles are shown (rows). All positions considered (**a**) are filtered (**b**) to retain informative positions where at least half the accessions cover the position with at least one read and at most a total of 300 reads, and where there are at least two reference and two alternative genotypes. Insertions and deletions are discarded, and only biallelic SNPs are kept. The succession of genotypes at a position is called variation pattern. Patterns are clustered through pair-wise comparisons (**c**) and compressed (**d**) to form a variation fingerprint. Similar variation patterns are retained if they occur at least at three genomic positions within the segment. To be considered a similar pattern they must have no differences in homozygous variants and can have up to 10% differences in their heterozygous variants. Fingerprints of all segments in the genome are compared to each other to find shared variation patterns between different segments (**e**)

To process comparable genomic units, the assembled contigs are divided into segments of a fixed size and variation patterns are summarized and counted per segment (Fig. 2). Rare patterns (i.e., occurring only at one or two genomic positions within a segment) are removed, and the remaining summarized variation patterns are referred to as “fingerprint” per segment. The comparison between contigs is based on comparing such variation fingerprints, and contigs are ordered according to their shared fingerprints. Contigs are merged and/or arranged into scaffolds if physical evidence is available, for instance provided by supporting mate-pairs or long reads. Relying solely on physical evidence such as mate-pairs is often ambiguous due to large repetitive regions. These regions will either not be spanned by mate-pair reads or will be spanned by reads that have multiple alignment positions. Hence, haplotype-based information provides additional guidance and confirmation for scaffolding.

### Detection of shared variation fingerprints using *Arabidopsis* data

**Fig. 3:**
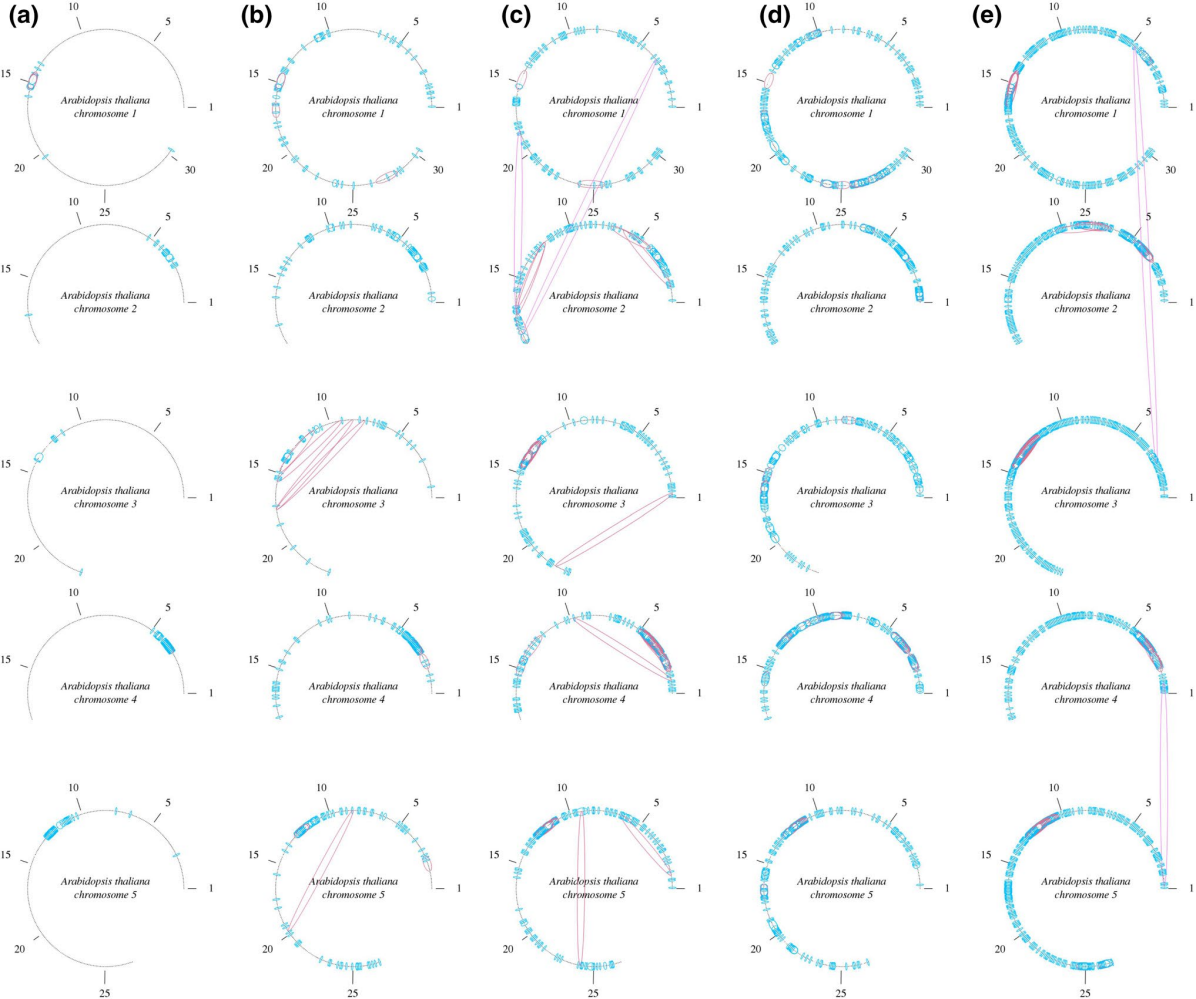
Haplotype connections in the *A. thaliana* genome obtained with different datasets. Each dot represents a 100-kbp genomic segment. Blue links: intra-chromosomal relations; red links: inter-chromosomal or intra-chromosomal relations with distance > 1 Mbp. Chromosome coordinates are displayed in Mbp at intervals of 5 Mbp. Relations are based on SNP datasets with minor allele frequency ≥ 0.05. Each arc represents an *A. thaliana* chromosome where relations are shown based on haplotype information with 15 (**a**), 30 (**b**), 50 (**c**) and 100 (**d**) randomly sampled accessions. The last sampling (**e**) is based on 50 northern Swedish accessions and is also the one with the highest density of haplotype-based relations

As a proof of concept, we took advantage of previously generated genotyping data based on whole-genome sequencing of 1135 accessions of the model plant *Arabidopsis thaliana* (Alonso-Blanco et al. 2016). In the context of that project, single-nucleotide variation had been determined relative to the reference sequence of the *A. thaliana* Columbia (Col-0) genotype. Based on this variation information we determined variation patterns per position in the Col-0 reference assembly and generated variation fingerprints per segment of 100 kbp. The number of shared fingerprints between such 100 kbp segments was compared for sets of 15 to 100 randomly chosen accessions out of the 1135 available datasets (Table 1). With as few as 15 randomly selected accessions we found a genome-wide average of three shared fingerprints per megabase confirming the assembly (i.e., located in adjacent segments) and up to 0.30 shared fingerprints per megabase between remote segments not supporting the assembly structure (Table 1, Online Resource 1). Most of the segments that were correctly identified as being adjacent based on shared fingerprints were located in centromeric regions. When using 100 randomly selected accessions the number of shared fingerprints in adjacent segments increased to 16 per megabase distributed along the whole genome, with at most 1.90 remote relations per megabase. Using genotyping datasets from 50 randomly selected accessions resulted in 11–13 adjacent shared fingerprints and 1.4–2 remotely shared fingerprints per megabase. Thus, collecting haplotype information based on 50 re-sequenced accessions appeared as a good trade-off between the number of sequenced individuals, the number of fingerprints relating adjacent segments to each other correctly, and the false-positive rate of remote relations (Fig. 3).

**Table 1:** Subsets of *A. thaliana* populations used for haplotype-based scaffolding of the *A. thaliana* Col-0 reference genome

			Relations per Mbp	
Number of accessions	Selection	Variant positions	Adjacent segments	Remote segments
15	Random	2,503,290	2.70	0.10
15	Random	2,470,324	3.27	0.28
15	Random	2,542,885	2.44	0.30
30	Random	3,477,787	7.87	0.66
30	Random	3,474,355	7.34	0.67
30	Random	3,311,788	6.73	0.32
50	Random	4,290,816	9.23	1.94
50	Random	3,890,368	11.93	1.35
50	Random	4,383,184	10.54	1.81
100	Random	5,358,002	14.85	0.98
100	Random	5,661,692	15.63	1.18
100	Random	5,367,110	14.60	1.90
15	Sweden (south)	2,174,936	7.69	0.78
50	Sweden (south)	3,248,749	18.73	2.94
50	Sweden (north)	2,222,345	23.19	3.36
50	Sweden (mixed)	3,228,322	18.30	3.61
50	Italy (north)	3,800,608	8.01	3.48

Total number of variant positions and average number of adjacent relations per Mbp as determined based on shared variation patterns reflect the genetic divergence in the population subsets

Next, we assessed the impact of the genetic relatedness of the accessions on the detection of adjacent genomic segments based on shared fingerprints. To do so, we selected accessions from two distinct geographic areas instead of the random selection. Fifty Italian accessions showed a density of adjacent shared fingerprints (11.5 per megabase) that was similar to randomly chosen ones. However, we observed more than twice as many (21.9) adjacent shared fingerprints per megabase when choosing 50 accessions from Sweden (Table 1). The set of Swedish accessions comprised samples from all over the country (North and South) whereby the northern accessions displayed the lowest local pairwise genetic distances of any *A. thaliana* accessions sampled in Europe and also had the highest rate of rare alleles in the context of the whole 1135 accessions dataset (Alonso-Blanco et al. 2016). Southern Swedish accessions had higher pairwise distances compared to northern ones but also had fewer rare alleles. Accessions from northern Sweden were thus the ones that shared the most haplotypes. This was also the dataset with the best performance in terms of density of haplotype-based relations. Italian accessions were chosen based on close geographical proximity within northern Italy but are known to cluster with different populations distributed throughout Eurasia (Alonso-Blanco et al. 2016). The close genetic relatedness among Swedish accessions was reflected in the number of variants: there was less variation between Swedish *A. thaliana* accessions relative to the Col-0 reference when compared to randomly sampled accessions (Table 1). For example, there were 2.2–3.2 million variant positions in 50 Swedish accessions versus 3.9–4.4 in 50 randomly sampled accessions. This underlines the importance of the presence of conserved haplotypes over larger genomic distances rather than merely a high number of variants to increase the amount of adjacent shared fingerprints.

### Quinoa genome assembly

We employed haplotype information for scaffolding of a de novo assembly of the quinoa genome which is about ten times larger than the genome of *A. thaliana.* In large repeat-rich genomes, contig ordering and scaffolding becomes a challenging task.

As a reference genome, we sequenced the genome of the Bolivian quinoa accession CHEN125, a typical white-seeded cultivar originally collected at Lahuachaca in the Bolivian Altiplano region at an altitude of 3800 m. Sequencing was performed on the Pacific Biosciences RS II sequencing platform, resulting in a dataset with an average read length of 10,464 bp and 65-fold genomic coverage (Table 2) assuming a haploid genome size of 1.45 Gbp (Kolano et al. 2012). The read length distribution had two peaks at 6800 bp and at 16,000 bp (Online Resource 2). The raw data were corrected and assembled using the Canu pipeline (Koren et al. 2017) resulting in an assembly size of 1.32 Gbp consisting of 6747 contigs with an N50 length of 608 kbp (referred to as "Qpac" assembly). Fifty percent of the Qpac assembly was comprised within 546 contigs (Table 3). Of a set of 2121 highly conserved plant genes used as input for BUSCO (Seppey et al. 2019; Simão et al. 2015) we found 2050 genes (96.7%) within Qpac, suggesting comprehensive coverage of the gene space in our assembly (Table 4). Of the 2050 BUSCOs, 184 were found in more than two copies (up to six copies) and 1287 BUSCOs appeared exactly twice. Close to 70% of BUSCO genes were duplicated, which reflects the tetraploid nature of the quinoa genome. Indeed, despite assembling like a diploid genome, quinoa originated from the hybridization of two *Chenopodium* species. Homeologous sequences were successfully assembled separately, as reflected in the assembly size of 1.32 Gbp which corresponds to twice the haploid size of its parental genome donors. Consequently, most BUSCO groups were detected twice, because they are present in separate homeologous chromosomes.

**Table 2:** Raw data for de novo sequencing of *C. quinoa* CHEN125 and re-sequencing of 50 quinoa accessions

Accession	Protocol	Read type	Reads [million]	Peak size [kbp]	Sequenced bases [Gbp]	Genome coverage
CHEN125	PacBio	Subread	8.6	6.8, 16.0	90.3	64.5*x*
	Nextera	Mate-pair	101.9	3.6	5.1	3.5*x*
			103.8	5.8	5.2	3.6*x*
			100.2	8.1	5.0	3.5*x*
	Illumina		296.4	2.5	14.8	10.2*x*
			312.2	4.2	15.6	10.8*x*
50 accessions		Paired-end	2958.3	avg. 0.5	369.8	avg. 5.1*x*

The column “peak size” refers to subread lengths (PacBio), span sizes (mate-pairs, 2 × 50 nt) and insert sizes (paired-ends, 2 × 125 nt), respectively. For short-read sequences, peak sizes were determined after alignment to the quinoa genome assembly Qpac. Genome coverages were estimated assuming a quinoa genome size of 1.45 Gbp. “avg.” means on average per accession

**Table 3:** Assembly metrics for Qpac (assembled by Canu) and RefCHEN125 (after haplotype-based scaffolding) and the metrics of other existing quinoa assemblies (ASM168347v1: Jarvis et al. 2017; Cq_real_v1.0: Zou et al. 2017; Cqu_r1.0: Yasui et al. 2016)

	Qpac	RefCHEN125	ASM168347v1	Cq_real_v1.0	Cqu_r1.0
Assembly size (Gbp)	1.320	1.314	1.333	1.337	1.087
N50 (kbp)	608	1079	3844	1159	87
L50	546	285	102	373	3768
Longest sequence (Mbp)	7.3	10.5	23.8	5.4	0.64
Largest contig group (Mbp)	–	39.7	–	–	–
Sequences ≥ 2 Mbp	68	125	217	95	0
Number of sequences	6747	5689	3487	3185	24,845

Genomic adjacency determined by haplotype information was used for contig scaffolding and merging

**Table 4:** BUSCO analysis of the Qpac quinoa assembly

	BUSCO groups	
	Number	%
Total	2121	100
Complete	2031	95.8
Complete (single copy)	560	26.4
Complete (duplicated)	1471	69.4
Fragmented	19	0.9
Missing	71	3.3

We mapped Illumina paired-end data from the same accession to the PacBio assembly and found that 450,353 sites were heterozygous (indels and SNPs included) corresponding to 0.034% of the total genome.

We estimated the genome size of quinoa accession CHEN125 based on the sequencing data by taking into account the k-mer frequency distribution. Based on 17-mers, we estimated a diploid genome size of 1.22 Gbp (Online Resource 11). Similar results were obtained for estimations based on *k* = 19 to *k* = 27 (between 1.23 and 1.27 Gbp). Quinoa is an allotetraploid plant, yet its subgenomes are sufficiently different so that its genome essentially behaves like a diploid (Schiavinato et al. 2021). The estimated genome size of 1.22 Gbp and the assembly size of 1.32 Gbp were in good agreement.

### Assembly integration with a public genetic linkage map

In order to assign our assembled contigs to the 18 quinoa chromosomes we downloaded sequences that had been previously used as markers for generating a genetic map of the quinoa genome based on single-nucleotide variation (Maughan et al. 2012) and located them in the Qpac assembly. Of 511 available markers specifying 29 linkage groups, 356 aligned uniquely to Qpac contigs. In total, 282 Qpac contigs comprising 270 Mbp (20.5% of the Qpac assembly) could be unambiguously assigned to a linkage group. Of these contigs, 225 contigs carried one marker and 57 contigs carried between two and five markers (Online Resource 3). The fraction of chromosomally assigned contigs could be substantially extended after re-sequencing a set of 50 publicly available quinoa accessions for variant calling and haplotype detection: When haplotypes spanned several contigs, connections to yet unassigned contigs could be established (see below).

### Sequencing of 50 quinoa accessions and variant calling

We selected 50 quinoa accessions from Bolivia and Peru originating from the vicinity of Lake Titicaca for low-coverage whole-genome sequencing (Online Resource 4). Seed material of these accessions is available in public repositories so that accessions can be re-grown if needed. Sequencing of genomic DNA was performed on the Illumina platform resulting in on average 32 million read pairs per accession, translating into an average of 5.7-fold genomic coverage (range 4.3-fold to 7.4-fold) before filtering. Quality filtered reads (mean coverage fourfold to sevenfold) from accessions were aligned to the reference genome at an average mapping rate of 47 to 59% (aligning uniquely as pairs within the expected insert size to the genome). The initial variant calling before further filtering using Qpac as reference resulted in a total of 5,005,967 variant positions of which 596,685 were insertions or deletions (indels) and 4,409,282 were single-nucleotide polymorphisms (SNPs) (Online Resource 5**)**. These were 1.44 million variants per accession on average of which 62% were homozygous and 38% were heterozygous. For comparison, we applied the same approach on a publicly available quinoa assembly of Chilean cultivar QQ74 (Jarvis et al. 2017), referred to as ASM168347v1, yielding 594,194 indels, 4,706,603 SNPs, and 1.89 million variant positions on average per accession (68% homozygous, 32% heterozygous). Considering that the ASM168347v1 assembly is based on a quinoa cultivar from Chile, while the Qpac reference and the re-sequenced samples originated both from the Bolivian and Peruvian Altiplano region, a higher degree of variation of the re-sequenced lines relative to ASM168347v1 was expected. The slightly lower amount of indels in ASM168347v1 could be explained by lower mapping rates to this genome (0.5–1% less) and more reads mapped as single reads (i.e., 1–2.5% more reads that could not be aligned as pairs because of one of the mates not mapping within the expected insert size range). This could indicate structural variation that is hard to detect with short Illumina paired-end sequencing data.

The transition/transversion ratio of variants detected using either reference had the same value of 1.50. The five accessions with the highest number of variants were the same for both genome references and originated from the Acomayo Province (Peru). Acomayo is located in the Cusco region that shares a border with the Ayacucho region where the domestication of quinoa is assumed to have occurred 5000 years ago (Lumbreras et al. 2008). The average density of variation of 3.34 positions per kbp (CHEN125) to 3.53 positions per kbp (QQ74) in the quinoa genome is comparable to variant densities in other domesticated plants (Xu et al. 2019) and is much lower than in wild plants such as *A. thaliana* (101.95 positions per kbp) (Alonso-Blanco et al. 2016).

There were 3,589,483 variant positions in Qpac when considering only biallelic SNPs covered by at least half of the 50 individuals and with minor allele frequency > 5%, which was the set of SNP data used for further analysis in the context of haplotype-based scaffolding. With these settings we observed 1.18 million variant positions on average per accession of which 61% were homozygous and 39% were heterozygous.

### Ordering of contigs based on haplotype information

**Fig. 4:**
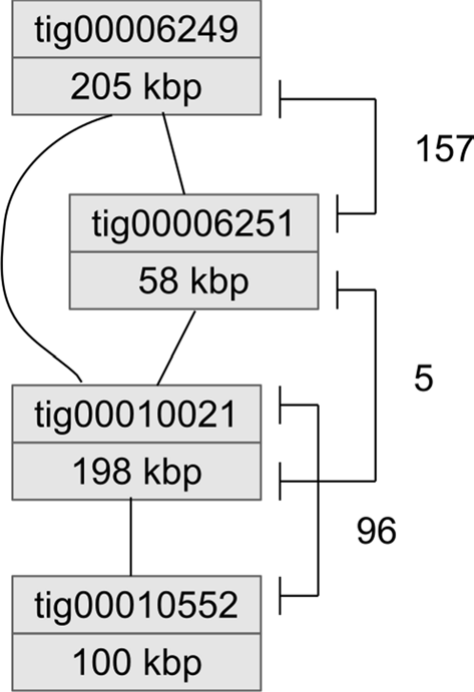
Order and orientation of four Qpac contigs based on haplotype information and physical evidence. Lines between contigs indicate a relation of high confidence based on shared variation patterns. Mapping of mate-pairs (spanning lines on the right, number of mate-pairs is indicated) confirmed the adjacency of the contigs and revealed their final order and orientation; tig00010021 had to be reversed according to the matching location of the mate-pairs. The resulting scaffold size was 561 kbp of sequence plus 28 kbp of gaps

The variants along the assembled contigs of Qpac were analyzed in segments of 100 kbp in size. Each contig smaller than 100 kbp (4390 contigs totaling 185 Mbp) was considered a single segment. In total, we obtained 15,204 such segments for our 1.32 Gbp assembly. For each segment we determined a variation fingerprint and performed an all-against-all comparison of fingerprints per segment. Some segments did not display variation and thus had no fingerprint. There were initially 12,366 segments distributed over 3172 contigs showing shared variation patterns with at least one other segment. Highly abundant patterns were removed as well as patterns that were supported only by few positions within a segment (presumably occurring due to rare mutations, recent mutations or wrong variant calls). Fingerprints between segments had to share at least 10% of their variant patterns to be considered as a sufficiently supported haplotype-based relation. After filtering out weakly supported relations of segments there were 2188 contigs that shared variation fingerprints with at least one other contig. Such contigs formed 371 groups consisting of two to 82 contigs (Online Resource 6). In total, the 2188 contigs represented 907 Mbp, i.e., 69% of the assembly. Since variation fingerprints may span several contigs, the local order of contigs was not always resolvable. Among the 371 groups, 306 formed small connective networks that could be unambiguously resolved into a linear structure (Fig. 4). These 306 groups contained 849 contigs (2–11 contigs per group) and encompassed 350 Mbp. Networks of the remaining 65 groups comprising 1339 contigs were more complex (Online Resource 7). To obtain the final assembly RefCHEN125 we only scaffolded or merged contigs for which actual physical evidence was available, i.e., which had their haplotype-based order supported by linking mate-pairs, long reads or overlapping contig ends (see below). The contigs that could not be physically linked remained within a contig group (reflected in the name of the assembly sequences). In these cases, haplotype information does not allow the determination of the exact order of contigs, but merely provides information about their adjacency. By our definition, contig groups may consist of *bona fide* scaffolds, but may also contain contigs without precise positional information.

Among the groups connected by variation fingerprints, 123 contained contigs previously anchored to genetic markers (Online Resource 6, Online Resource 3). There was general agreement between the genetic markers and the groups formed by variation fingerprints. For example, five among 38 contigs extending the 3.1 Mbp contig “tig00000000,” previously anchored to linkage group 2 (LG 2), carried genetic markers all of which were consistently from LG 2. Of the total of 282 genetically anchored Qpac contigs (270 Mbp), there were 214 contigs that could be extended by 1206 additional contigs (452 Mbp) via shared variation fingerprints (Table 5). Remaining groups not anchored to genetic markers encompassed 250 Mbp in 768 contigs within 248 groups.

**Table 5:** Fraction of Qpac contigs that could be related to other contigs using haplotype information and/or could be anchored to linkage groups using genetic markers

	Number of contigs	Mbp	% of Qpac assembly
Related anchored	1420	658	50
Related unanchored	768	250	19
Orphan anchored	68	65	5
Orphan unanchored	4491	347	26
Total	6747	1320	100

In summary, of the 1.32 Gbp quinoa Qpac assembly, 908 Mbp showed improved ordering after exploiting shared variation fingerprints (i.e., haplotype information). The remaining 4559 contigs (411 Mbp) with a mean size of 90 kbp lacked variation so that fingerprint comparisons could not be performed. However, of these, 68 contigs in 65 Mbp had already been chromosomally anchored based on genetic markers. The total genetically anchored fraction of the assembly after considering haplotype information was 722 Mbp comprising 55% of the Qpac assembly.

### Verification of haplotype-based relations

The order of contigs based on haplotype information was verified based on consistency with Illumina mate-pairs, long reads, and/or overlapping contig ends, in comparison with another quinoa assembly, and by the assignment to linkage groups via genetic markers.

Among the 123 contig groups that also contained at least one genetic marker, only three groups contained markers from two different chromosomes. This was the case for Qpac contig “tig00007425” (965 kbp) previously assigned to LG 1 but related to several contigs assigned to LG 6 by haplotype information. However, “tig00007425” also contained a uniquely aligning genetic marker from LG 6 with a lower mapping score (Online Resource 3). The LG 6 marker Cq04033_520 mapped close to the left end of the contig and the LG 1 marker Cq07822_1014 mapped near to the right end of the contig. All contigs related via variation fingerprints and assigned to LG 6 emerged from the left end of “tig00007425.” Upon inspection of the read coverage we noticed a coverage drop between the two markers, indicating the possible location of a misassembly in “tig00007425.” Similar cases were found in the two other groups containing contradictory linkage group assignments; these groups also showed coverage drops between contradictory markers (“tig00005716” assigned to LG 12 grouped with four LG 15 assigned contigs and “tig00008618” assigned to LG 3 grouped with two LG 10 assigned contigs). Thus, apart from confirming available LG assignments, the haplotype information led to the identification of three cases of potential misassemblies which were resolved by splitting the respective contigs. On the other hand, 36 haplotype-based contig groups contained at least two genetic marker from the same LG and confirmed each other.

The group comprising the largest number of contigs included two LG 1 contigs and a total of 82 contigs (13 Mbp in total, Online Resource 6). The largest group in terms of basepairs included 13 contigs on LG 5 and encompassed a total of 74 contigs (40 Mbp). This group was also the one with the highest number of contigs containing genetic markers, i.e., 13 markers in total. Overall, the organization of contigs based on haplotype information confirmed the LG assignments based on genetic markers.

**Fig. 5:**
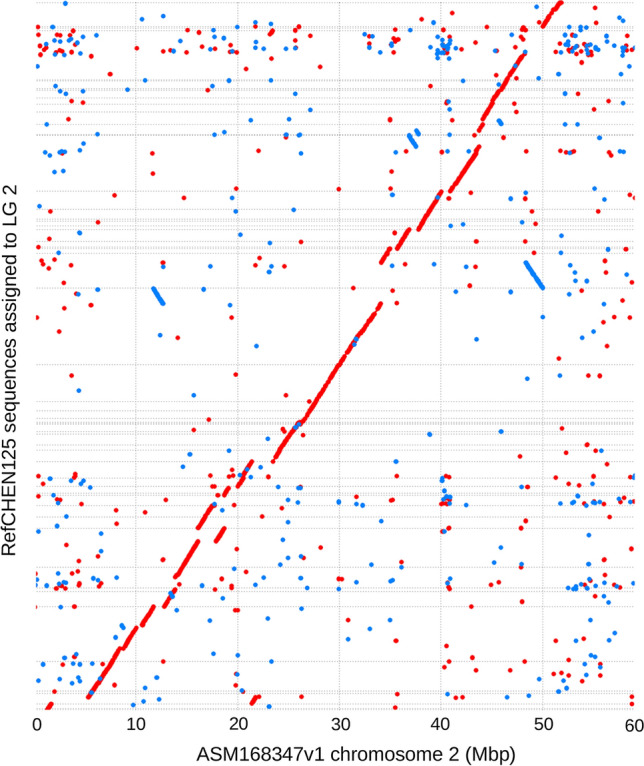
Nucmer alignment between RefCHEN125 sequences assigned to quinoa chromosome 2 and the ASM168347v1 chromosome 2 assembly. Blue lines depict large-scale inversions, and red lines depict large stretches of full-length alignment between the two assemblies

The contig groups were overall in agreement with the chromosome-level quinoa assembly ASM168347v1 from the Chilean cultivar QQ74 (Jarvis et al. 2017). For example, when aligning all contigs and contig groups assigned to LG 2 (44 Mbp in total) to the ASM168347v1 assembly, most of them had an end-to-end sequence alignment in chromosome 2 (Fig. 5). The ASM168347v1 chromosome 2 is 59 Mbp in length, including 2 Mbp of unspecified bases. While networks created by haplotype information could not fully determine the order and orientation of each contig, the overall order of larger contigs and their chromosome assignment were consistently confirmed by ASM168347v1. The scaffolding of CHEN125 permitted the highlighting of three large-scale inversions in chromosome 2 when compared to QQ74, which were strongly supported by paired-end data (Fig. 5, blue lines).

To further validate the haplotype-based contig ordering and orientation we sequenced Illumina mate-pairs of five different span sizes ranging from 2.5 kbp to 8.1 kbp (Online Resource 8). We also took overlaps between contigs and spanning long reads into account. Among all the haplotype-based relations (6435), 3351 had at least one supporting long read. Long reads had a size similar to the largest mate-pairs library insert size (about 10 kbp) so that connections between contigs overlapping by more than 10 kbp or between contigs that had misassembled ends longer than 10 kbp could not be spanned by the reads. In total, we connected 1,848 contigs into 790 sequences containing two to 13 contigs. In total, 1,298 connections between contigs were confirmed by mate-pairs, 416 connections were confirmed by overlaps, and 712 connections were confirmed by both mate-pairs and overlaps. Additionally, long reads confirmed 1480 connections already supported by mate-pairs or overlaps. Overall, among the 2188 contigs related by haplotype information 84.5% had confirming physical evidence in the form of bridging mate-pairs, matching long reads, and/or overlaps to establish their connections.

### Scaffolding and merging of quinoa contigs

We integrated information from overlaps, mate-pairs, and long reads to improve the contiguity of the Qpac assembly and obtained a scaffolded version called RefCHEN125. The RefCHEN125 assembly had an N50 length of 1079 kbp, a total size of 1.314 Gbp, and encompassed 5689 sequences (Table 3). The difference in size between RefCHEN125 in respect of Qpac was mainly due to contig overlaps supported by mate-pairs or long reads. Because of the span sizes of our mate-pair libraries (max. peak size 8 kbp) that were in the same range as the PacBio subread length, the detected links resulted more often in merging than in insertion of gaps. The main improvement arose from 2188 contigs being reordered according to their adjacency in the genome inferred from haplotype information. The Qpac assembly initially provided contiguous information for a maximal stretch of 7.3 Mbp, whereas RefCHEN125 provided haplotype-based evidence for ordered contigs in large regions of up to 39.7 Mbp and scaffolded contigs in regions of up to 10.5 Mbp. The sequences in the FASTA file of RefCHEN125 were ordered and renamed to reflect their position within the assembled genome.

## Discussion

In this work, we show how the continuity of haplotypes in the genome can be exploited to improve genome assemblies. Initially, we used variant data generated during the 1001 *Arabidopsis* genome project (Alonso-Blanco et al. 2016) to assess the validity of our approach. Thereafter, we newly calculated a genome assembly based on PacBio long-read sequencing data for the crop plant quinoa and scaffolded the assembled contigs by exploiting haplotype information. Haplotypes were determined based on variation analysis of a set of fifty quinoa cultivars that we sequenced during this study. Additional data types such as long-range short-read pairs were used to confirm the haplotype-based contig ordering.

The non-random association between pairs of alleles, called linkage disequilibrium (LD), can be measured using *r*^2^, the square of the correlation coefficient. As we expect to find longer continuous haplotypes when there is high correlation between pairs of alleles, populations with lower LD decay are better suited for haplotype-based scaffolding. We plotted the LD decay of the *A. thaliana* populations and the set of quinoa individuals used in this study (Online Resource 10). In the sampled datasets of *A. thaliana*, the population of mixed Swedish individuals had both the most haplotype-based connections and the lowest LD decay rate, i.e., the correlation between pairs of alleles was the highest over genetic distance. Furthermore, the LD decay profiles of the other thale cress datasets were in agreement with the amount of haplotype-based relations that we found, i.e., the faster LD decay was, the fewer haplotype-based relations we found. The quinoa population used to construct haplotype-based relations had a promising LD decay profile. Indeed, despite the relatively low amount of variation in the quinoa genome, we could establish many haplotype-based connections because the correlation between loci was higher than in any of the thale cress datasets. This was to be expected given that quinoa is a self-fertilizing crop that will maintain LD over hundreds of kilobasepairs. Nevertheless, it needs to be stressed that re-sequencing data for quinoa had rather shallow coverage of 5.7-fold in our work. Such low coverage may result in suboptimal variant calling, and using genomic re-sequencing data at higher coverage may lead to more precise variant calling, and, hence, better haplotype-assisted genome assembly scaffolding. However, we demonstrate that a low sequence coverage is sufficient to detect haplotype-based relations as we evaluate the combined coverage of all accessions. A locus showing variation only in a few accessions would be discarded both due to low sequencing coverage and due to a small minor allele frequency in the studied population. Indeed, alleles with a low minor allele frequency are less relevant for haplotype-based relations because they are rarely haplotype markers. In summary, while a low sequencing depth generally complicates the analysis of variant calling and the detection of haplotype-based relations, it can be compensated if the studied population is large enough. In quinoa, 50 accessions were sufficient to obtain haplotype-based relations that substantially improved the assembly.

### Distribution of haplotype-based relations

Most genomes exhibit an uneven distribution of recombination events (Dohm et al. 2014; Mackiewicz et al. 2013). High recombination rates are observed within the chromosome arms in proximity to the telomeres, while recombination is infrequent in centromeric regions. In *A. thaliana* chromosomes, centromeric regions coincide with regions of dense haplotype-based relations indicating a high degree of haplotype conservation and are among the few regions that show haplotype relations based on genotyping data from only 15 randomly chosen accessions. Several centromeric characteristics can explain such results. The peri-centromeric regions of chromosomes in *Arabidopsis thaliana* and in its relative *Arabidopsis lyrata* exhibit highly conserved gene order, indicating that there is very little crossing-over in this region (Kawabe and Nasuda 2005). Thus, centromeric haplotypes are not disturbed by repeated recombination events and are more conserved over generations resulting in more reliable variation patterns. These peri-centromeric regions also display higher variation rates which help distinguishing haplotype blocks with as little as 15 accessions, so that variants to rely on are abundant. At the centromere itself we find no haplotype-based relations because there is actually very little variation reported in the variant catalog provided by the 1001 *A. thaliana* genome project (Alonso-Blanco et al. 2016). We can hypothesize that regions with high variant density, but little shared variation are regions where the haplotypes are disturbed by frequently occurring recombination events. In other words, due to the high frequency of recombination, haplotype blocks are so small that shared variation between contigs is undetectable with genotyping information. Additionally the high SNP density of the *A. thaliana* genome permitted extensive benchmarking of the scaffolding methodology exploiting haplotypes. Despite the fact that haplotype block sharing seems to have more impact than the amount of variant positions, the success of the method will depend on SNP density to some extent. This can explain why in the quinoa genome the number of haplotype-based relations was scarcer than in *A. thaliana.*

### Impact of relatedness of accessions on the method

There is less total variation in southern Swedish *A. thaliana* accessions relative to the Col-0 reference when compared to randomly sampled accessions (total number of 2.17 million variant positions for 15 southern Swedish accessions and 2.5 for 15 randomly sampled accessions). However, using genotyping data only from Swedish accessions permits detecting more haplotype-based relations outside of centromeric regions in comparison with randomly chosen accessions. This indicates that the issue does not lie in the amount of variation but rather in the conservation of haplotype blocks over larger genomic regions, which seems to be the case when focusing onto accessions sampled within a confined geographic range with a shared evolutionary history. Genotyping datasets generated from individuals with a cumulatively higher number of variants will not necessarily yield better results. What seems to be important is the relatedness between the used individuals, i.e., extensive sharing of haplotypes. The number of haplotypes contained in a group of individuals is dependent on its evolutionary history, but a continuity along the genomes is expected. In a mapping population each individual has continuous regions of its genome belonging to either parental haplotype. We showed that in a loosely related population such relationships between haplotype number and genomic location exist, and the more closely related a population is the easier such patterns can be observed. Using this characteristic for assembly improvement is a first step, but we can see how such information could help describe the evolutionary history and relationship of a group of individuals as well.

### Haplotype sharing across large distances

During our study, we noticed the existence of variation patterns that are shared between regions far apart in the genome, either on the same but also on different chromosomes. In other words, there are haplotypes which get co-inherited even though the regions are not physically linked and therefore, in theory, could freely recombine. These associations are found frequently in both the *A. thaliana* genome and in quinoa. For example, based on 50 randomly sampled *A. thaliana* accessions, 36% of the 1,193 segments used for fingerprint building contained such haplotypes. They were also previously identified in *Drosophila* and human genomes (Curtis and Vine 2010; Curtis 2016; J Zhang et al. 2003) and nicknamed “yin-yang” haplotypes because they differ at each SNP position. For the purpose of scaffolding genome assemblies, positions supporting such mismatching haplotypes were often filtered out because they heavily overlapped with variation patterns widespread throughout the genome: in the above-mentioned *A. thaliana* sampling, after filtering for highly re-occurring patterns, 10% of the segments carried such “yin-yang” patterns. This type of filtering can be done in the context of a high-quality genome assembly, because other variation patterns derived from the flanking regions can be used to detect adjacencies. On the other hand, such interactions could reflect functional genetic interaction, i.e., epistasis, even though, aside from selection, other scenarios such as random drift or genetic admixture may be taken into consideration when interpreting such findings (Zan et al. 2018).

###  Value of the method

Several options exist to convert genome assemblies into arrangements of scaffolds that approximate pseudochromosomes. Popular concepts for long-range genome assembly scaffolding are based on Hi-C or Chicago data (Putnam et al. 2016), linked reads (Zheng et al. 2016), or optical mapping (Lam et al. 2012). A drawback of these strategies is their requirement for high-quality, high-molecular weight DNA,since DNA of such quality often is not available for plant samples. Genome assembly scaffolding exploiting haplotypes is insensitive to DNA quality, because re-sequencing can be performed even on degraded samples.

In recent years, genome de novo assembly has moved on so that nowadays long-read data are preferentially used to reconstruct the sequence of genomes. However, general assembly problems remain also in long-read assemblies. First, breaks are often introduced when contig extension stalls due to ambiguities in contig elongation. In practice this means that many pairs of contigs exist in an assembly which in fact overlap. Integrated with haplotype information, such cases can be identified followed by contig merging. Second, misassemblies can occur. Haplotype information supports misassembly detection, as variation patterns will change globally at a given position where contigs got wrongly connected. By additional inspection of local read densities, the misassembly can be localized in order to split the contig. We have shown that haplotype information allows grouping of contigs and scaffolding in project setups where multiple accessions get sequenced, leading to variant catalogs. For the current project, only single-nucleotide variants were used. However, we expect that small Indels could be very informative as well for haplotype-based scaffolding. Using variant catalogs to improve genome assemblies is attractive both cost-wise as well as scientifically, considering that the value of a genome assembly increases with its contiguity.

Similar concepts were developed that use different approaches, like LDMAP and LDscaff (Pengelly and Collins 2019; Zhao et al. 2020). LDMAP splits genomic regions in terms of linkage disequilibrium units, whereas we use intervals with fixed sizes. Our approach finds haplotype-based relations between all intervals of the region used in LDMAP example data when using relaxed parameters and between almost all intervals when using parameters similar to those used in our other datasets. Both LDMAP and LDscaff are sensitive to breakpoints between regions with rapidly decaying linkage (i.e., recombination hotspots) and thus rely on detecting these breakpoints. Lack of variation is not a huge drawback for LDMAP or LDscaff. On the contrary, our approach relies on the presence of many variants and is less sensitive to loss of linkage disequilibrium because we do not rely on the presence of linked alleles directly but rather on a similar pattern of variation at a locus in a population, i.e., even a small number of similar patterns is sufficient to detect a haplotype-based relation. However, removing alleles with a small minor allele frequency is crucial in order to reduce noise. We may assume that allele frequency-based methods and our method complement each other as they rely on different aspects of the data resulting in different contig connections.

## Methods

###  Plant material and DNA extraction

As the quinoa reference genotype we chose the Altiplano accession CHEN125. Together with fifty additional quinoa accessions from different localities in Bolivia and Peru, seeds were obtained from the Genebank of IPK Gatersleben (http://www.ipk-gatersleben.de/en/gbisipk-gaterslebendegbis-i/). Plants were grown under greenhouse conditions. High-molecular weight DNA for sequencing of accession CHEN125 on the SMRT Pacific Biosciences (PacBio) RS II sequencing platform was obtained from grinding freshly harvested leaves under liquid nitrogen into powder, followed by extraction of the genomic DNA using a Nucleospin XL kit (Macherey–Nagel, Düren, Germany). DNA from leaf material of further accessions was isolated using a streamlined CTAB protocol (Saghai-Maroof et al. 1984), followed by one step of chloroform extraction. After precipitation, DNA was dissolved in 1 × TE (Tris–EDTA) pH 8.0.

###  Sequencing

Long-read sequencing was performed at the Max Planck-Genome Centre in Cologne, Germany, using the PacBio RS II sequencer with P6-C4 sequencing chemistry (Pacific Biosciences, Menlo Park, CA, USA), using size selected libraries with insert of 15 kbp or larger. The run time was 360 min. For the project, a total of 107 SMRT cells were used with an approximate output of 800 Mbp of sequencing data in terms of polymerase reads per SMRT cell. Genome re-sequencing was performed with Illumina technology (Illumina, San Diego, CA, USA). Two-hundred nanograms of genomic DNA quantified using a Qubit fluorometer (Life Technologies, Foster City, CA, USA) were sheared to a peak size of 580 bp with a Covaris M220 Focused Ultrasonicator (Covaris, Woburn, MA, USA). Thereafter, genomic sequencing libraries were prepared using a TruSeq Nano LT library preparation kit (Illumina) using indexed adapters. The quality and quantity of the libraries were assessed on a Bioanalyzer 2100 instrument (Agilent, Santa Clara, CA, USA) using a DNA 1000 chip. Eight libraries with different barcodes were pooled into one sequencing lane, aiming at five-fold genomic coverage for each sequenced accession. Sequencing was done at the Vienna BioCenter Core Facilities (VBCF), Vienna, Austria, on the Illumina HiSeq 2500 instrument using v4 sequencing chemistry and a 2 × 125 nt paired-end sequencing recipe. Additional Illumina sequencing (2 × 50 nt) of the reference genotype CHEN125 was done to generate mate-pair data: two libraries with span sizes of 2.5 kbp and 5 kbp were prepared using a modified protocol based on the Illumina PE-112–2002 kit (Dohm et al. 2014), and three libraries (span sizes of 4 kbp, 6 kbp, 9 kbp) were prepared using Illumina Nextera technology.

### Genome assembly

The raw PacBio SMRT reads of quinoa genotype CHEN125 were processed using the Canu pipeline v1.3 (Koren et al. 2017). We required that all reads were corrected by Canu using the default settings for SMRT data (-pacbio-raw) and with an expected post-correction error rate of 0.01. We kept reads larger than 2 kbp after correction corresponding to 60-fold coverage of the quinoa genome. We assembled the data with Canu on a server with 128 GB memory on a SLURM grid engine accessing 14 cores with 2.60 GHz and 28 threads each and named the resulting assembly "Qpac". QUAST (Gurevich et al. 2013) and BUSCO v3 (Seppey et al. 2019) with the plant eudicotyledon set odb10 (https://busco.ezlab.org/) were used to assess contiguity and comprehensiveness, respectively, of the assembly.

### Genome size estimation

We used Illumina paired-end data to estimate the genome size of quinoa using a k-mer approach. We trimmed reads with trimmomatic (Bolger et al. 2014) and used jellyfish to sample 17mers from these high-quality sequences (jellyfish count -m 17 -o genome_17mer.jf) (Marçais and Kingsford 2011). We used jellyfish histo (jellyfish histo genome_17mer.jf -o genome_17mer.histo) and plotted the distribution of the 17-mer coverage. The estimated peak of the distribution was 81.4 (Online Resource 11). The sequencing depth of 17-mers was estimated at 99.3 using the formula seqDepth = kmerCovPeak*readLength/(readLength-kmerSize + 1), and the genome size was estimated at 1.22 Gbp using the formula Gsize = total read length/k-mer sequencing depth (as suggested by https://arxiv.org/abs/1308.2012). The total read length used for the experiment was 121,826,911,748 which is equivalent to a genome coverage depth of 84x. Illumina sequencing read length of the libraries used for genome size estimation was 100 nt, and the average post-trimming read length was 89 nt (readLength = 89 was used in the formula above). We also performed genome size estimations with k-mer sizes 19, 21, 23, 25 and 27.

### Read alignment

Raw paired-end Illumina reads from re-sequenced quinoa accessions were trimmed with trimmomatic version 0.35 (ILLUMINACLIP:adapters.fa:2:30:10 LEADING:25 TRAILING:25 SLIDINGWINDOW:10:25 MINLEN:36) (Bolger et al. 2014). Quality was assessed with fastqc (Andrews 2010). Trimmed reads were aligned to the Qpac assembly and to the assembly ASM168347v1 from the Chilean quinoa accession QQ74 (Jarvis et al. 2017) with bowtie2 (Langmead and Salzberg 2012). During read alignment we allowed for up to 12 mismatches or a gap length of up to 23 nucleotides or a mixture of both for a read of size 125 with maximum read Phred quality. We increased the amount of effort to extend the seed (− D 20), to find a non-repetitive seed (− R 3), and required a seed length of 20 (− L). We extracted the uniquely aligning read pairs using the AS and XS tags of the sam output. We kept reads with secondary alignments only if the difference between the primary (AS tag) and the secondary (XS tag) alignment score was larger than 10, meaning at least two mismatches for bases of maximum sequencing quality. Reads without reported secondary alignments were kept regardless of the alignment score. We kept concordantly paired reads (forward—reverse orientation, within expected insert size of minimum 300 and maximum 1000 nucleotides) if at least one of the reads aligned uniquely. We used picard (http://broadinstitute.github.io/picard/) MarkDuplicates with REMOVE_DUPLICATES set to “true” to remove PCR duplicates.

Long reads from Pacific Biosciences were aligned to the assembly to detect additional overlaps and contig connections. Reads were mapped with minimap2 (Li 2018) using default parameters. Orientation between pairs of contigs was determined only if reads mapped within 10% of the contig's head or tail. Some reads had been split during the correction phase of the Canu assembler; those split reads were also taken into consideration when finding connections as they belonged to the same original molecule. Out of the 6435 haplotype-based relations found, 3551 had at least one long read supporting it.

### Variant calling

To call variants we used samtools (Li 2011) mpileup to summarize the information contained in the alignment output and performed a bcftools variant call with − v (variants only) and − c (consensus caller) options followed by vcf-annotate hard filtering (–hard filter). We retained single-nucleotide variant (SNV) positions within the expected coverage range (− f MinDP 10 and − f MaxDP 300), without strand bias (− f StrandBias = 0.05 and at least one read on each strand per individual), without end-of-read bias (i.e., SNVs supported by ends of reads only, − f EndDistBias = 0.05) and with a support for the alternative allele call of at least 4 (− f MinAB = 4). We excluded SNVs within ten nucleotides surroundings of a gap (− f SnpGap = 10) and SNVs that appeared in clusters of three within the same ten nucleotides (− f SnpCluster = 3,10) as well as positions with less than 50% missing genotypes. These settings were used for the initial variant calling that was performed on Qpac and theQ74 ASM168347v1 assembly in order to compare the number of variants. For the analysis of haplotypes in Qpac we additionally applied a minor allele frequency of ≥ 0.05, considered only biallelic single-nucleotide polymorphisms, and selected positions showing less than 10% missing genotypes to establish and compare variation patterns (vcftools–maf 0.05–min-alleles 2–max-alleles 2 –remove-indels).

### Assembly integration with a quinoa genetic map

We located genetic makers previously used to construct a quinoa linkage map (Maughan et al. 2012) as sequences of 169–301 nt in length on the Qpac assembly by aligning them with blastn (Z. Zhang et al. 2000) (-evalue 1e-10 -word_size 50 -perc_identity 95). We kept markers with a unique alignment or with a distinct best alignment. Of 511 markers available, 451 had been assigned to defined linkage groups (LGs) with chromosome numbers by Maughan et al. (2012). Of these, 345 had unique mapping positions on Qpac. Further 18 non-uniquely mapping markers were added because they mapped on a contig containing a uniquely aligned marker of the same LG. Four markers had no match at all on the Qpac assembly (Cq06322_773 of LG2, Cq06528_1162 of LG6, Cq07623_1383 of LG7 and Cq09993_1704 of LG9).

#### A. thaliana datasets

We downloaded a variant call format (VCF) file from the *A. thaliana* 1001 genomes project (Alonso-Blanco et al. 2016) located at https://1001genomes.org/data/GMI-MPI/releases/v3.1/1001genomes_snp-short-indel_only_ACGTN.vcf.gz and generated subsets using vcf-subset.

### Linkage disequilibrium decay analysis

For the generation of the LD decay plot we used vcftools to downsample the datasets (vcftools–gzvcf dataset.vcf.gz–recode–recode-INFO-all–thin 1000–out "thin-dataset") and plink (http://pngu.mgh.harvard.edu/purcell/plink) with recommended parameters to calculate the LD decay using the r^2^ metric between the pairs of alleles in the downsampled data (plink –bfile "thin-dataset" –r2–ld-window-r2 0–ld-window 999,999–ld-window-kb 1000–out "thin-dataset"). Finally, we averaged the r^2^ obtained per distance with awk and plotted them with ggplot2.

### Pipeline for detecting haplotype-based relations

We developed a program called Haplopath which takes a variation catalog format (VCF) file as input and determines which genomic segments of the reference genome share variation patterns. Segments that share at least one variation pattern are considered as “related” whereby in subsequent filtering steps only those relations are extracted that are sufficiently supported. The program is coded in Perl v5.10 and is composed of the main script haplopath_connect.pl, the Perl module Patts.pm and the scripts haplopath_validator.pl and haplopath_viz.pl for filtering and visualization, respectively. Apart from Patts.pm Haplopath uses Perl modules that are usually available with a standard Perl distribution (Getopt::Long, File::Basename, Parallel::ForkManager, Storable, Data::Dumper, List::MoreUtils) and is multi-threadable. The key parameters of Haplopath are the segment size and maximum difference allowed between two positions showing variation (i.e., variation patterns). Further parameters are explained in the help of the program (–help). The segment size determines in how many slices the reference genome is processed. The variation patterns within each segment will undergo clustering and compression based on similarity (Fig. 2). Similarity depends on the succession of variant or reference alleles within the fixed order of individuals, heterozygosity level, amount of missing genotypes, and on the number of individuals. We call the collection of compressed variation patterns within a segment a “fingerprint.” The output of Haplopath is a plain text file and a Perl binary file containing the genomic fingerprints and the fingerprint-based relations. The binary file is used as input to the Perl script haplopath_validator.pl that can extract valid relations (i.e., supported by a sufficient number of variation patterns, see below) and format the relations in a list used as input for visualization with haplopath_viz.pl (see below).

#### Noise filtering

Haplopath performs an initial filtering step where uninformative positions are deleted. To keep a position, default settings assume ≥ 50% of accessions with genotype information, at least two accessions with the reference allele and at least two accessions with the alternative allele. Additional filtering is applied after the fingerprint building step: variation patterns that appear in more than 50 fingerprints (implying that more than 50 Mbp is spanned given a segment size of 100 kbp) are filtered out to avoid spurious connections due to recurrent common haplotypes.

#### Variation pattern comparison

The assembly is analyzed in evenly sized segments, and SNVs in each of those segments are extracted and stored separately. Default segment size is 100 kbp. Within a segment, variation patterns at each position are compared pair-wise, clustered and compressed based on similarity. The starting pattern for the comparison is updated for missing or heterozygous genotypes each time it finds another similar pattern. By default, two patterns are allowed to differ by 10%. This means that with 50 accessions, a maximum of five genotypes can differ either by missing values or due to heterozygosity. By default, no homozygous differences are allowed between two variation patterns. Also, in the case of very few alternative genotypes in the starting pattern, no genotype mismatches are allowed in those alternative allele accessions specifically (the same is valid in the case of very few reference genotypes). This threshold is set to 20% of the pattern’s length; in the case of 50 accessions if there are less than ten alternative genotypes, no mismatch is allowed in those accessions specifically. The starting pattern has an associated counter that keeps track of the number of similar patterns found within the same segment. The collection of compressed starting variation patterns (i.e., updated after each match) and its associated occurrence is the variation fingerprint of the segment.

All fingerprints are compared pairwise and the extent of the overlap (i.e., number of shared variation patterns) between them is documented as a Jaccard score (Hamers et al. 1989). The variation fingerprint does not have to be 100% identical, and a single shared variation pattern, regardless of its occurrence, is enough to document a relation in the program’s output. However, the associated similarity score based on the number of shared variation patterns between the two compared fingerprints is used to distinguish between strong and weak relations. If segment 1 (S1) has two variation patterns (P1 occurrence 5 and P1’ occurrence 10) and segment 2 (S2) has a single variation pattern similar to P1’ (P2 occurrence 9), then the similarity score is:

$$\frac{S1 \cap S2}{{S1 + S2 - S1 \cap S2}} = \frac{10}{{\left( {10 + 5} \right) + \left( 9 \right)*c - 10}} = 0.667$$

where *c* = *10/9*, the coefficient to even out the intersection between the two segments. Such a relation would be considered valid (≥ 0.1) and strong.

#### Visualization of the Haplopath output

We used the GraphViz module in Perl to visualize the haplotype-based adjacency as determined by Haplopath (program haplopath_viz.pl). The output of the visualization script is in dot language and interpreted by the circo or dot roadmap (Gansner et al. 2000) as a graph representing adjacency by connecting lines (edges) between contigs (nodes). Different implementations were used for *A. thaliana* and quinoa. Haplotype-based relations found in quinoa were visualized in separate graphs/figures.

#### Merging and scaffolding

The connection of contigs was performed using megamerger as implemented in the EMBOSS suite (Rice et al. 2000) for computing overlaps and by taking alignment information of mate-pairs and PacBio long reads into account. If mate-pair or long-read evidence and overlap evidence existed to confirm a haplotype-based relation between two contigs, mate-pair and long-read information was used preferentially. A stretch of Ns was introduced according to the distance estimated by mate-pairs. This distance was calculated using the cumulative distance from the alignments positions at the edges of contigs and subtracting the expected peak span size of the library. In case of an overlap, contigs were trimmed according to the coordinates indicated by megamerger and a stretch of 10 Ns was introduced. Orientation of contigs was determined based on mate-pairs, long reads and overlaps. Orientation provided by haplotype-based relations was only indicative (Online Resource 7) and cannot be directly trusted because shared haplotypes are not continuous in the collection of unrelated quinoa accessions. In the rare case of contradictory orientation obtained from the five mate-pair libraries, the orientation with the most supporting reads was chosen. On average 20 read pairs supported a haplotype-based relation that ended up in scaffolding or overlapping with mate-pair information.

#### Software and hardware specifications

Haplopath runs on Linux machines (tested on CentOS release 6.10 with 32 cores and 128 GB memory). It makes use of Perl libraries usually available with Perl 5.10 distributions. In addition it appeals to the system to use bcftools (view and query), vcf-annotate, vcftools, and sed.

## Supplementary Information

Below is the link to the electronic supplementary material.

Supplementary file1 (PNG 21 KB)

Supplementary file2 (PDF 3528 KB)

Supplementary file3 (PNG 40 KB)

Supplementary file4 (ODS 32 KB)

Supplementary file5 (ODS 26 KB)

Supplementary file6 (ODS 27 KB)

Supplementary file7 (ODS 28 KB)

Supplementary file8 (PDF 377 KB)

Supplementary file9 (PDF 21 KB)

Supplementary file10 (ZIP 828 KB)

Supplementary file11 (PNG 40 KB)

## Data Availability

The RefCHEN125 assembly is accessible at NCBI’s Genomes Database under the identification number JADNSA000000000. The raw data for de novo sequencing of CHEN125 are accessible at the NCBI Sequence Read Archive (SRA) under accession numbers SRR13014586 (PacBio subreads) and SRR13001694-SRR13001698 (Illumina mate-pairs). The SRA accession numbers for the Illumina paired-end datasets of genomic resequencing of 50 quinoa lines are listed in Online Resource 4. The code of Haplopath consisting of one Perl module (Patts.pm) and three Perl scripts (haplopath_connect.pl, haplopath_validator.pl, haplopath_viz.pl) is provided with this article (Online Resource 9) and is available at the github repository https://github.com/albodrug/haplopath. Filtered Qpac variants are available at https://bioinformatics.boku.ac.at/Download/ChenopodiumQuinoa/.
