# Supplementary figures and images for: Quinoa genome assembly employing genomic variation for guided scaffolding
Source: Theor Appl Genet. 2021 Aug 7;134(11):3577–94. doi: 10.1007/s00122-021-03915-x (PMC8519820; doi:10.1007/s00122-021-03915-x)

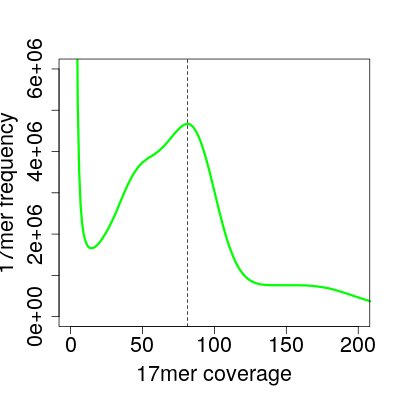

Supplement: Supplementary file 1 — Supplementary file1 (PNG 21 KB) [file 122_2021_3915_MOESM1_ESM.png]

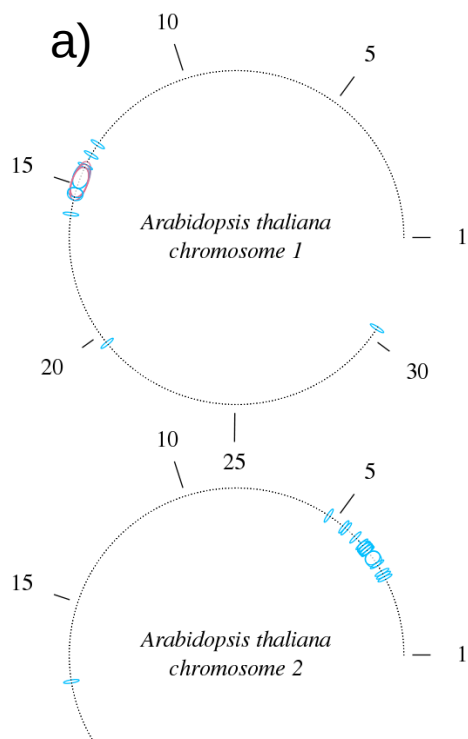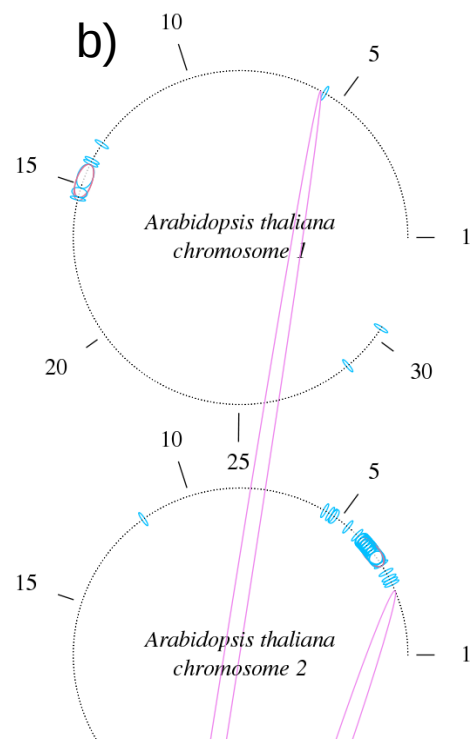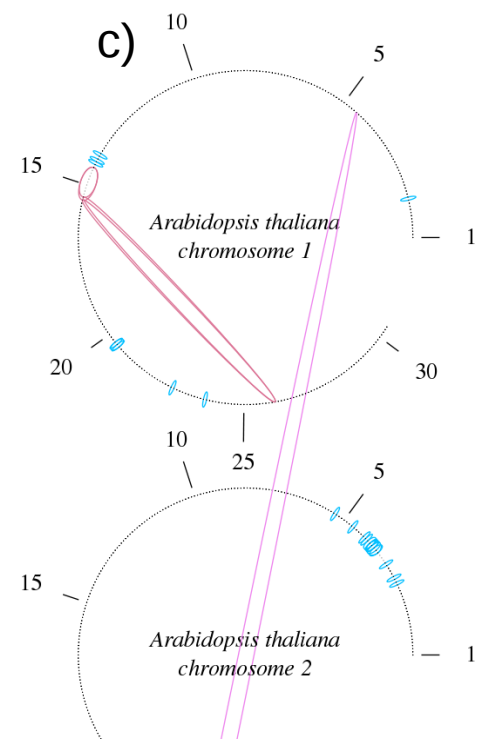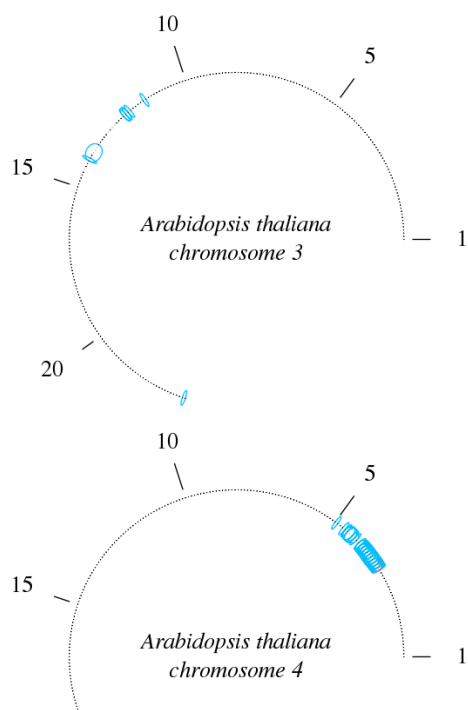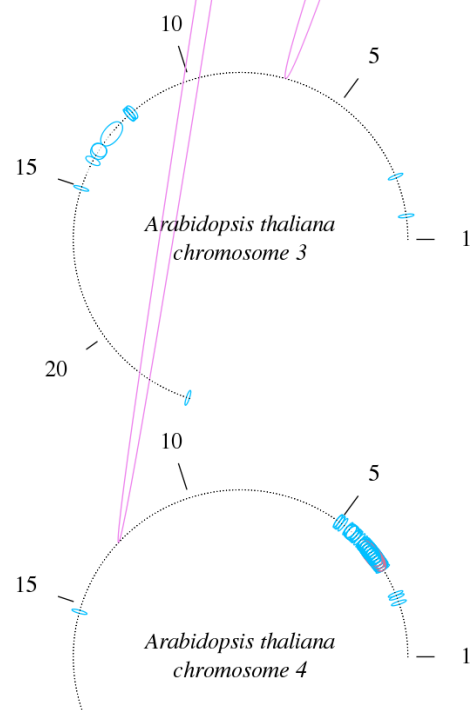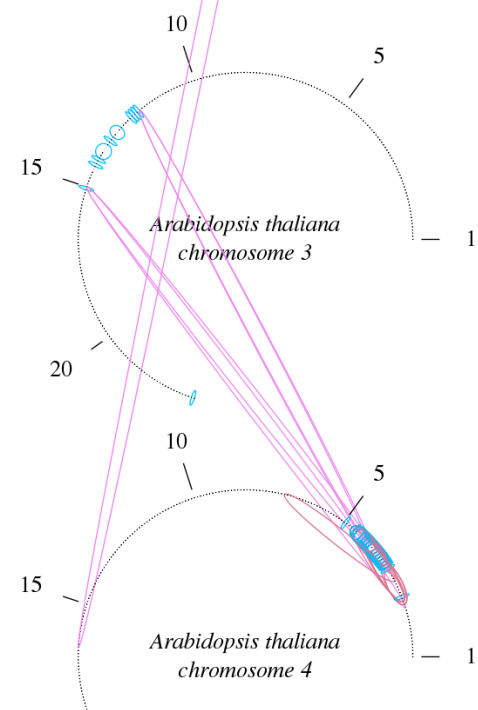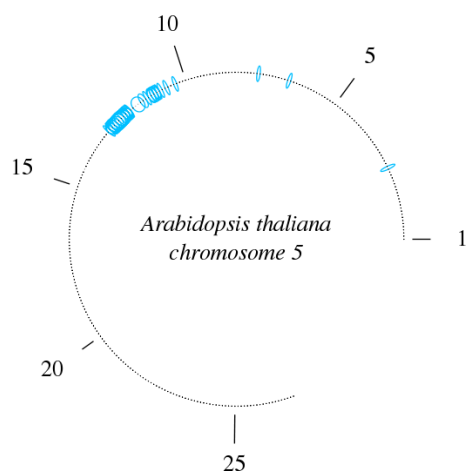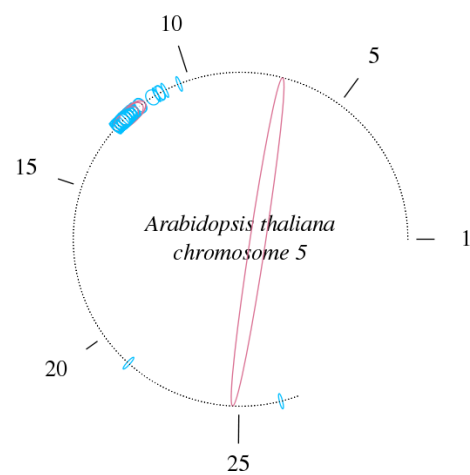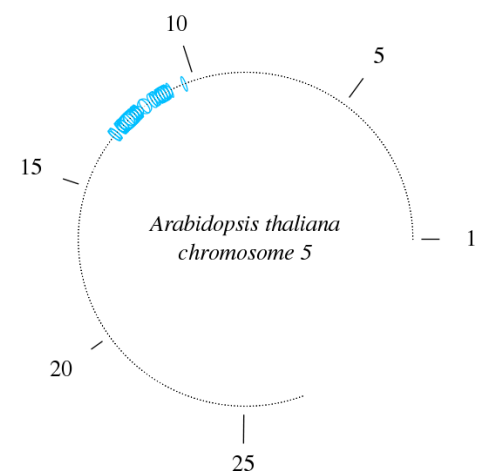

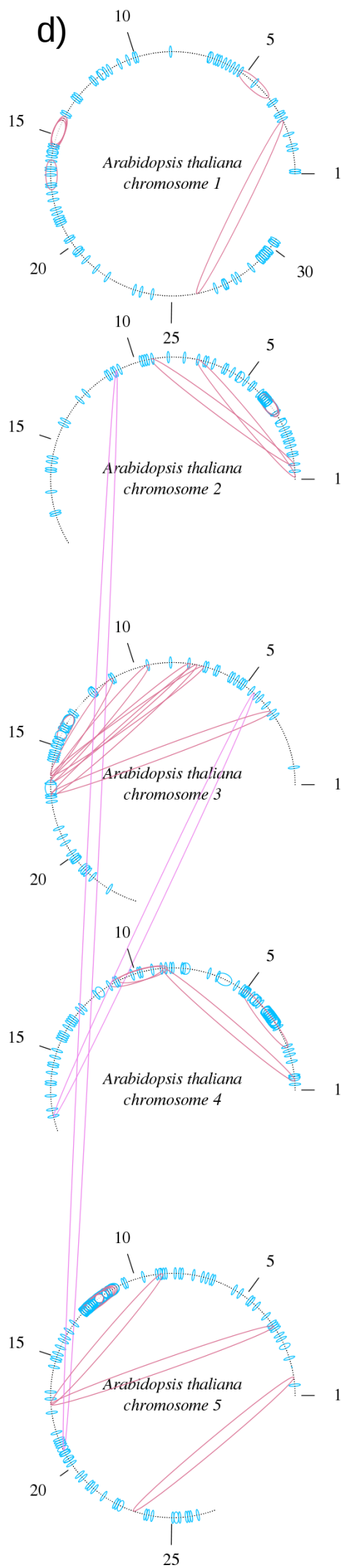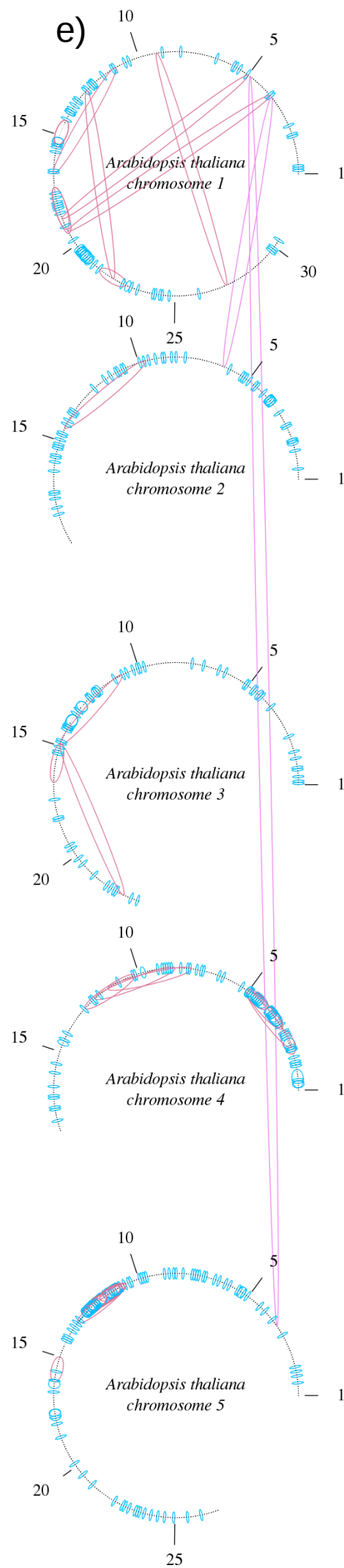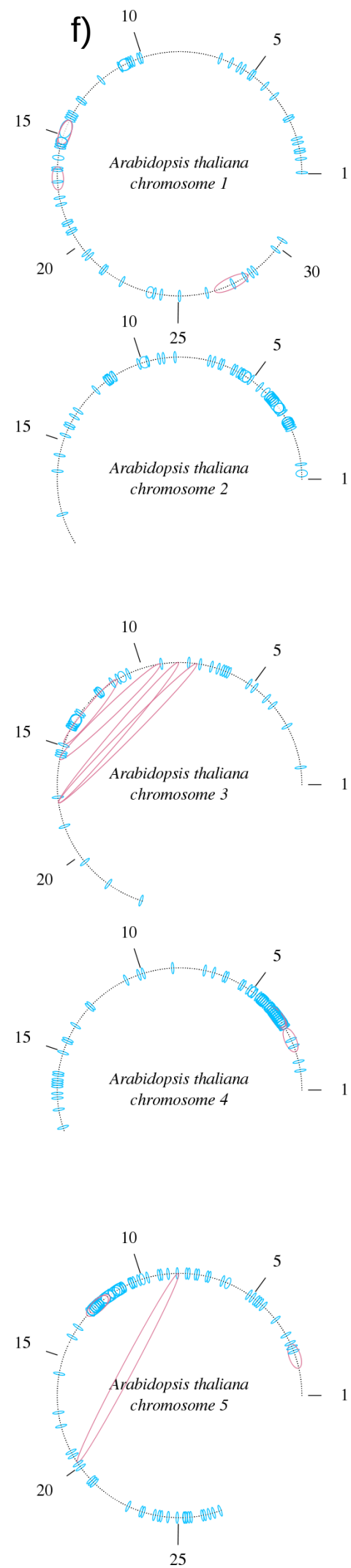

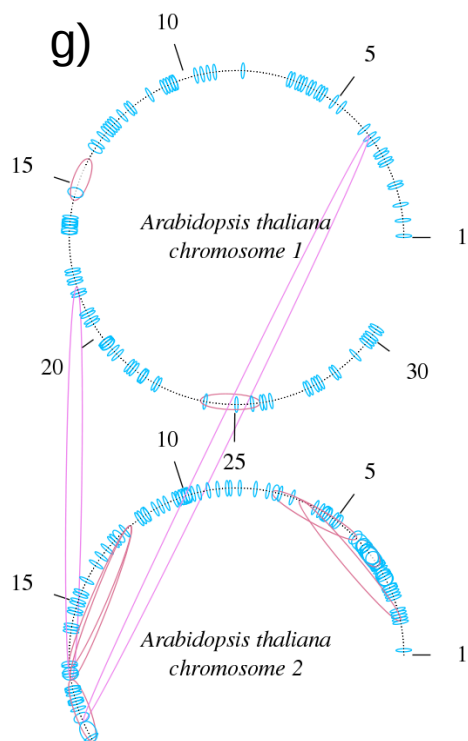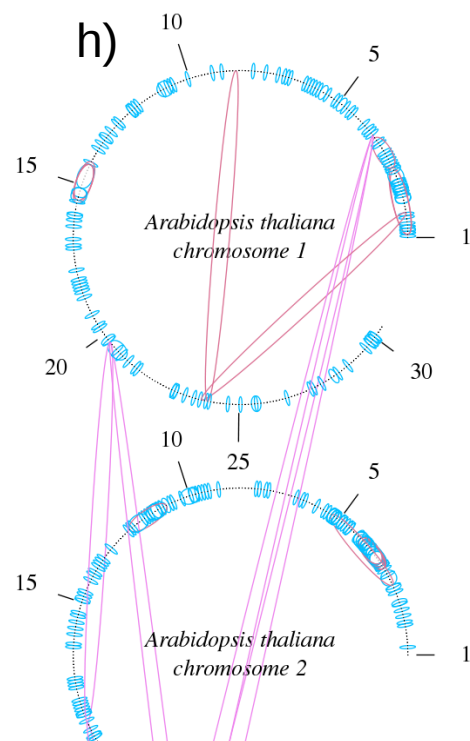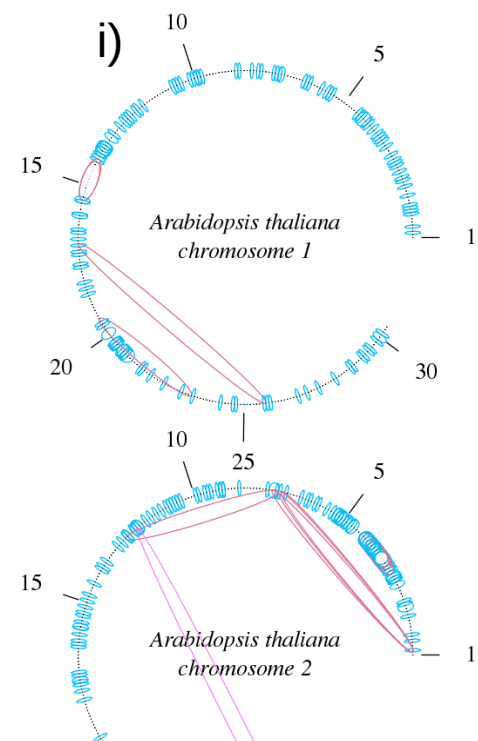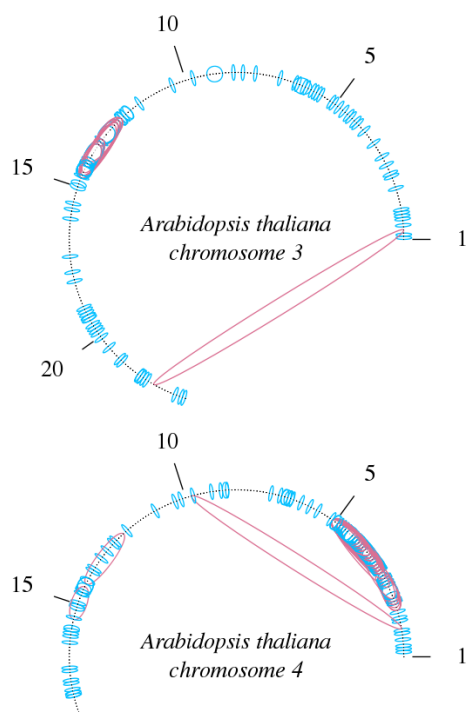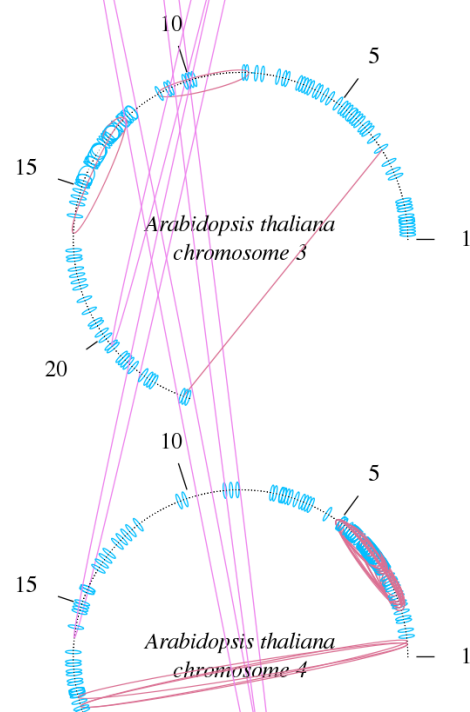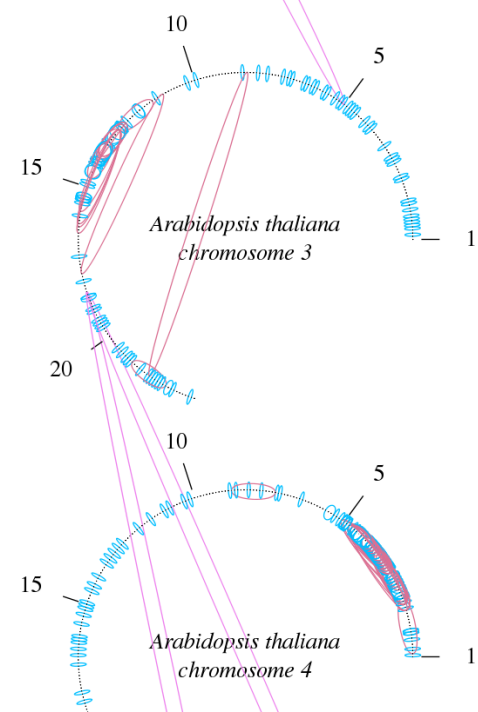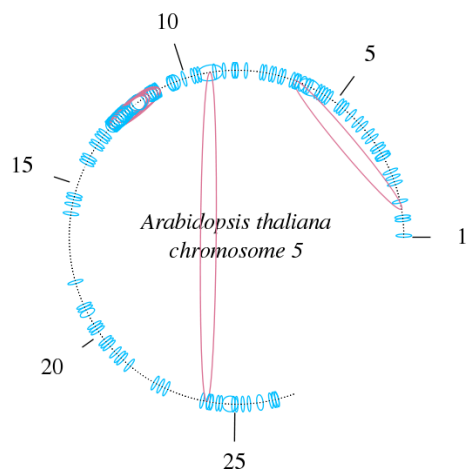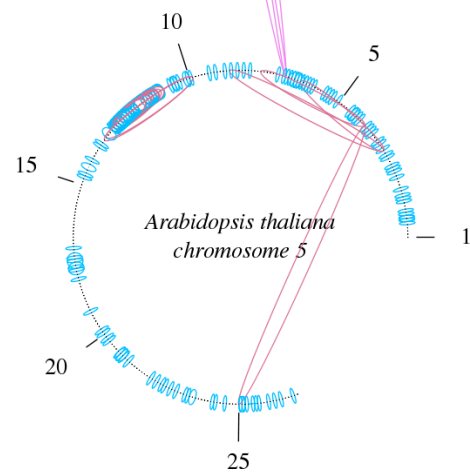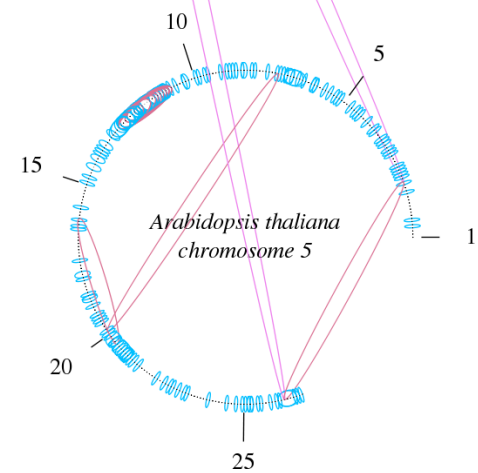

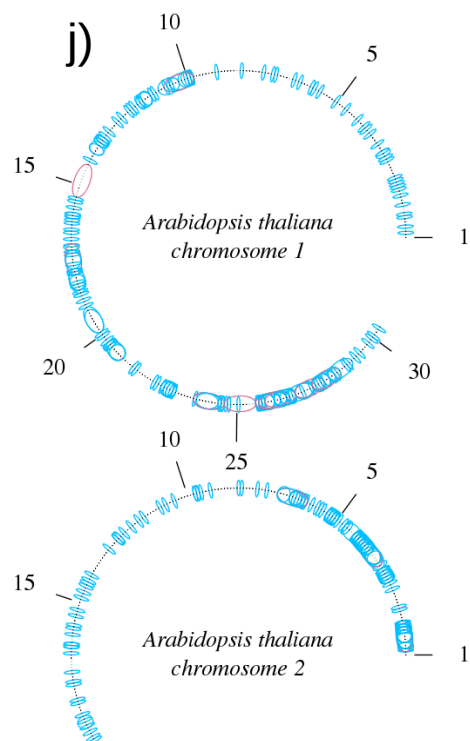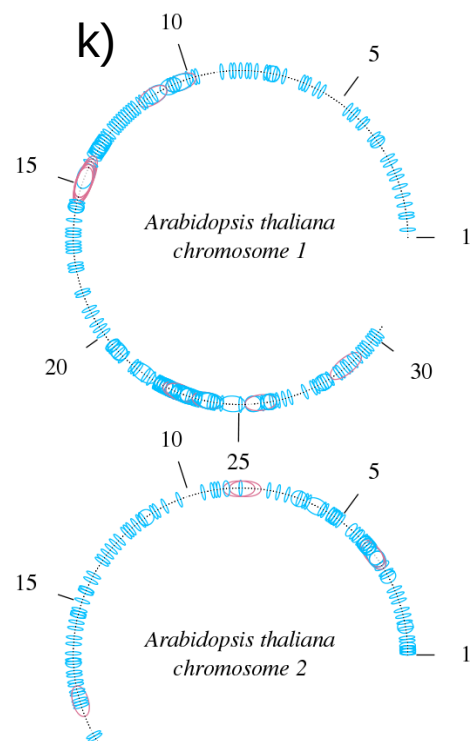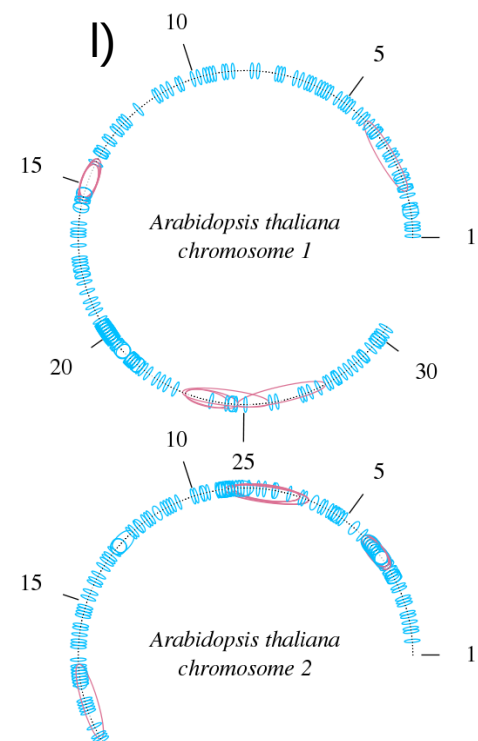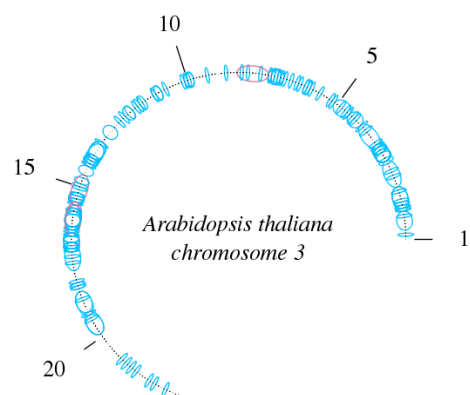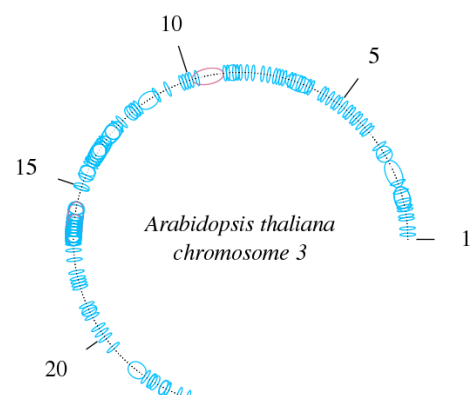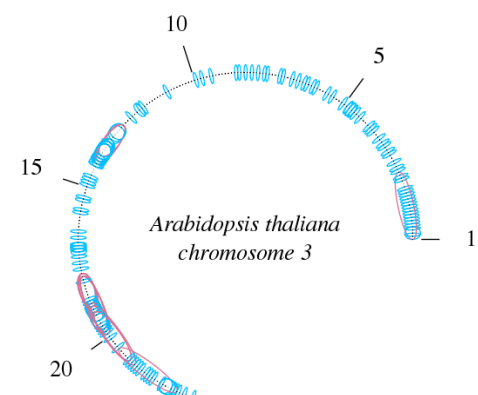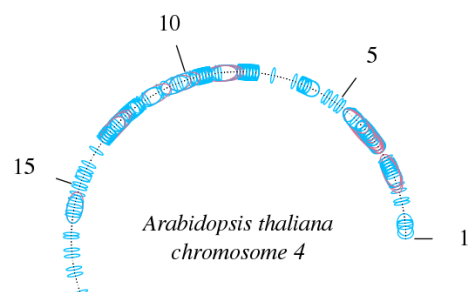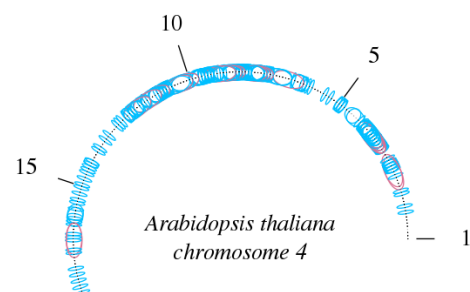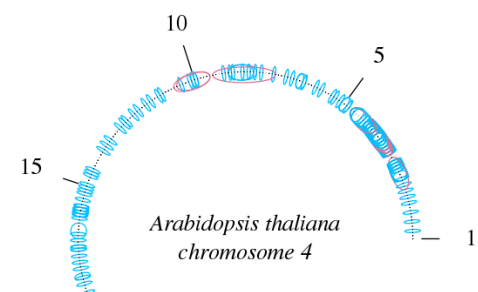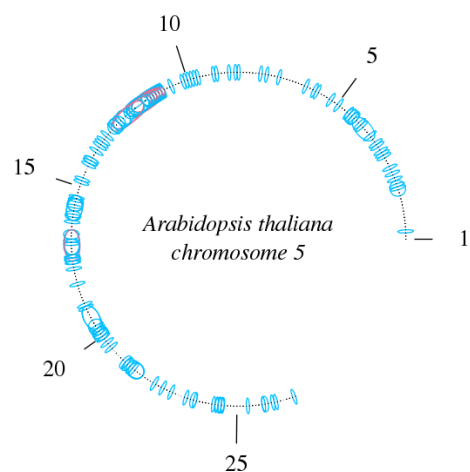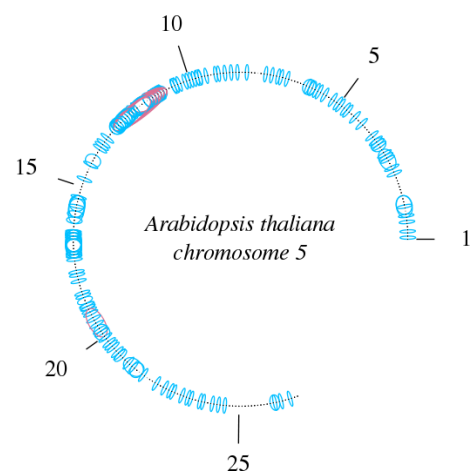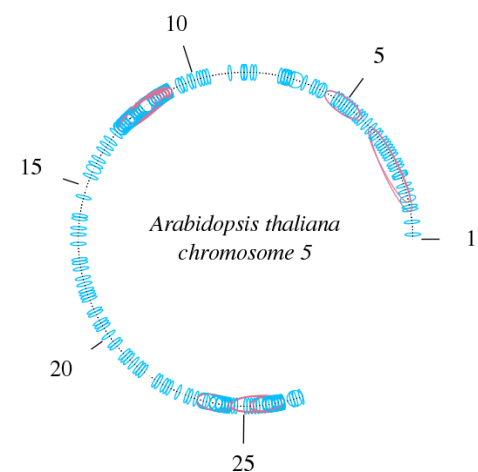

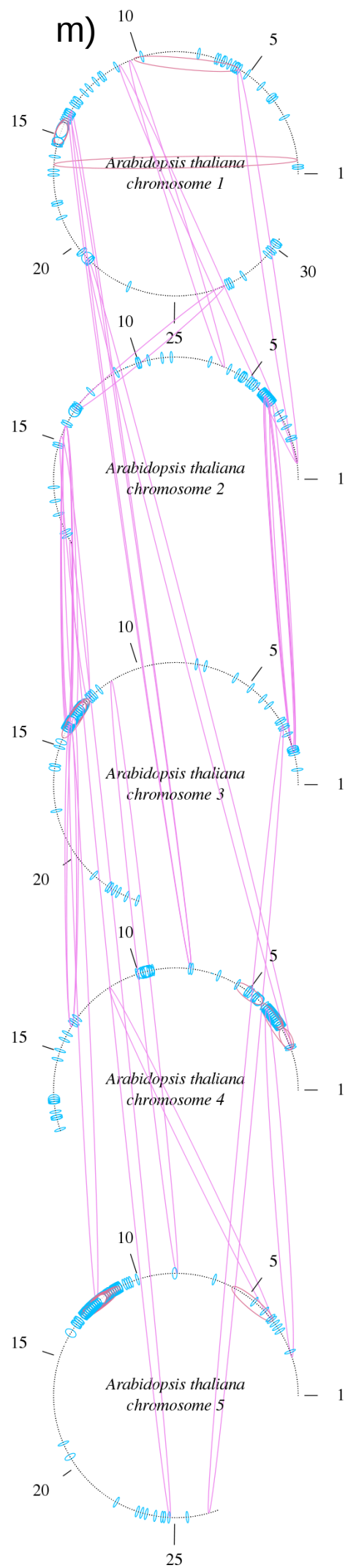

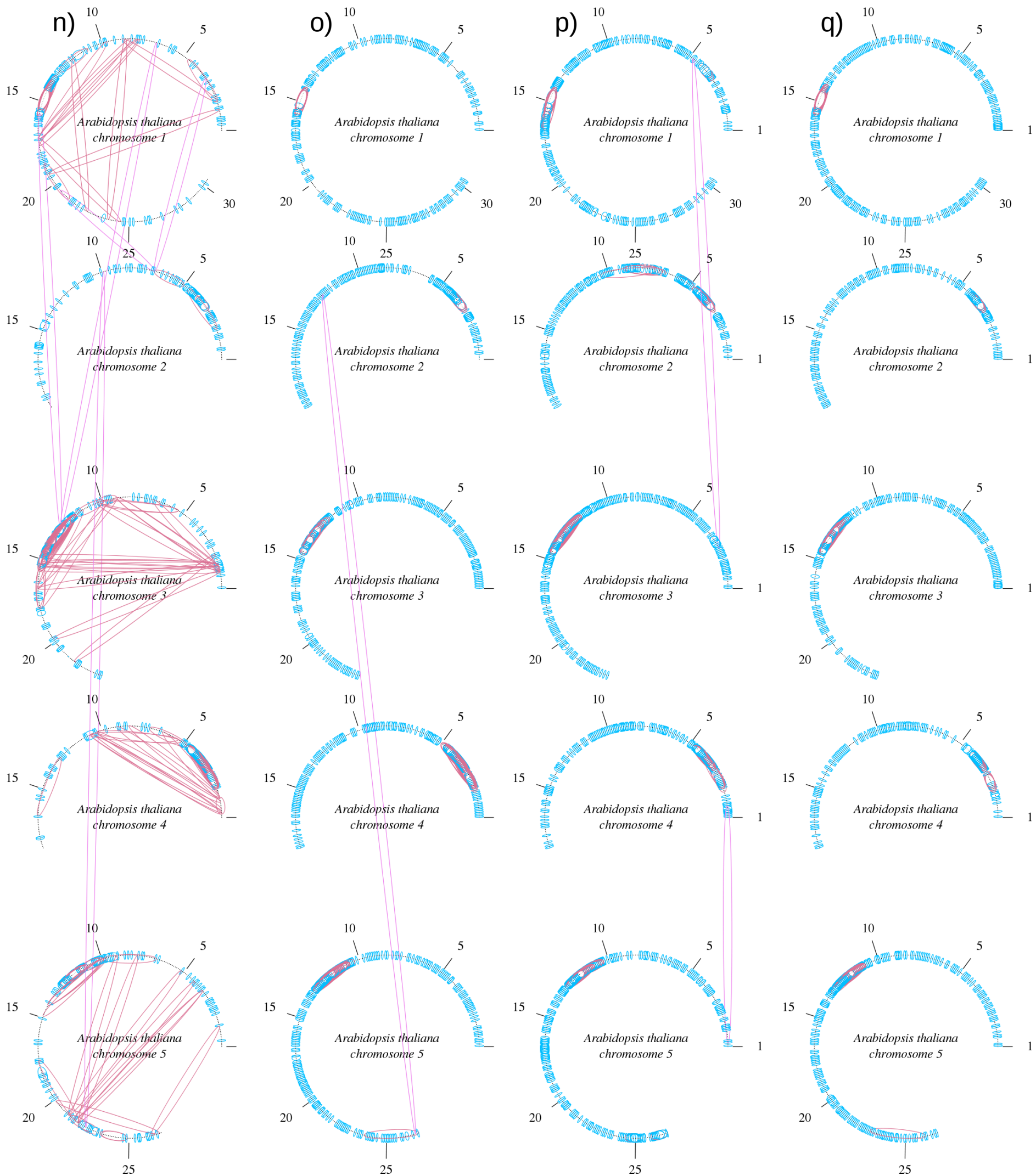

Supplement: Supplementary file 2 — Supplementary file2 (PDF 3528 KB) [file 122_2021_3915_MOESM2_ESM.pdf]

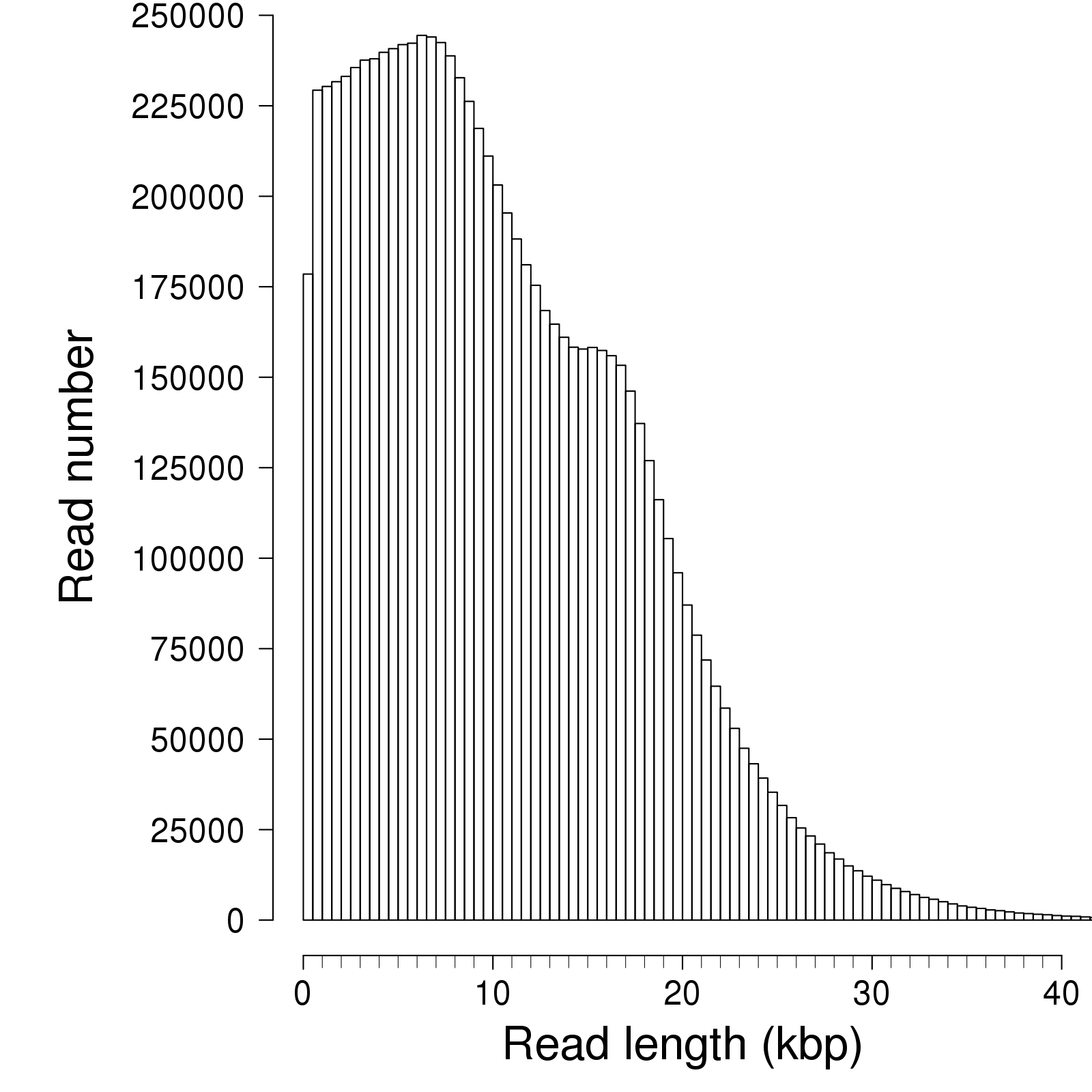

Supplement: Supplementary file 3 — Supplementary file3 (PNG 40 KB) [file 122_2021_3915_MOESM3_ESM.png]

a)

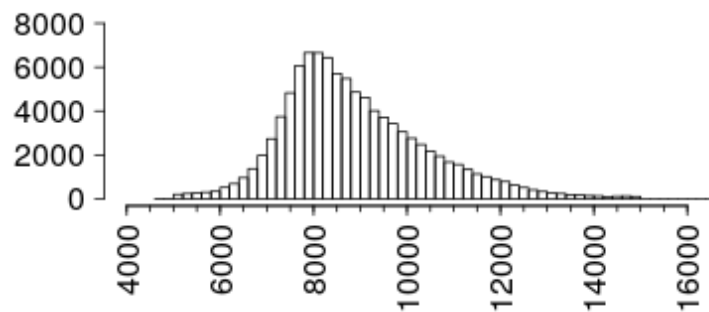

b)

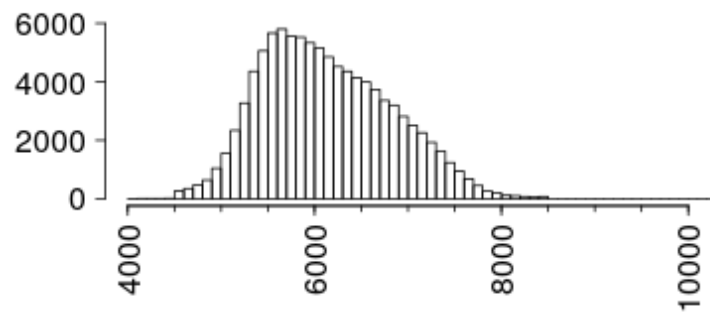

c)

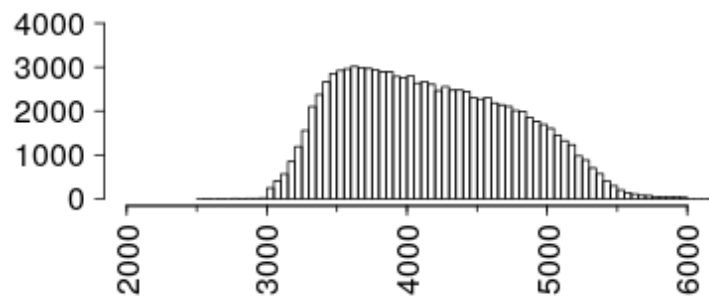

d)

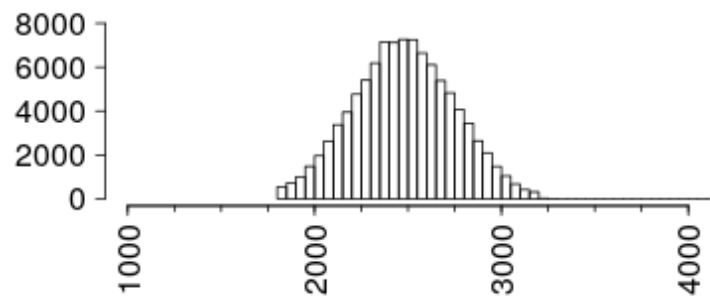

e)

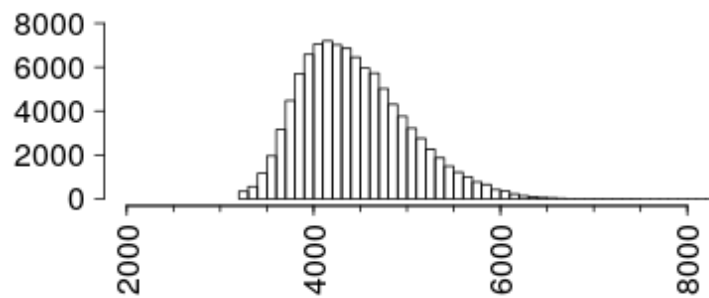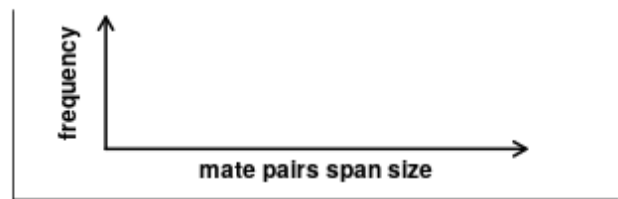

Supplement: Supplementary file 9 — Supplementary file9 (PDF 21 KB) [file 122_2021_3915_MOESM9_ESM.pdf]

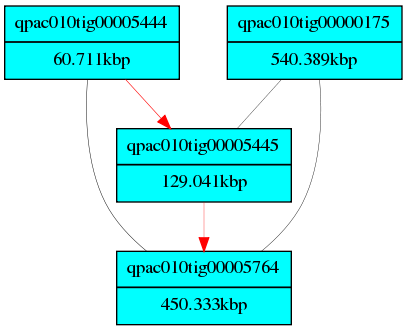

Supplement: Supplementary file 10 — Supplementary file10 (ZIP 828 KB) [file 122_2021_3915_MOESM10_ESM.zip › SupplFile-HAPLOCODE/code_varpat/test_output/sample_graph.png]

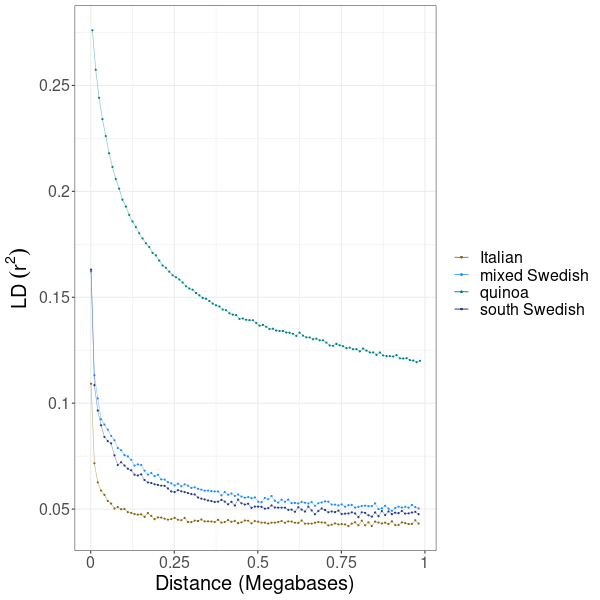

Supplement: Supplementary file 11 — Supplementary file11 (PNG 40 KB) [file 122_2021_3915_MOESM11_ESM.png]
