# Supplementary material for: Quinoa genome assembly employing genomic variation for guided scaffolding
Source: Theor Appl Genet. 2021 Aug 7;134(11):3577–94. doi: 10.1007/s00122-021-03915-x (PMC8519820; doi:10.1007/s00122-021-03915-x)
Supplement: Supplementary file 8 — Supplementary file8 (PDF 377 KB) [file 122_2021_3915_MOESM8_ESM.pdf]

# Examples of complex networks obtained with haplotype based connection solved with physical evidence (mate pairs, overlaps)

Contig carrying a genetic marker
Contig without a genetic marker
} Types of contigs

Haplotype based connection:
- tail-head
- head-tail
- tail-tail
- head-head
- undetermined

} Types of haplotype connections

Haplotype based connection confirmed by physical evidence

Haplotype based connection NOT confirmed by physical evidence (absence of evidence or contradicting evidence)

Scaffold or contig obtained with haplotype and physical evidence (if network obtained with haplotype connections results in more than one connected sequence).

} Confirmation by chysical evidence

1 Numbers indicate order of contigs within a scaffold.

a)

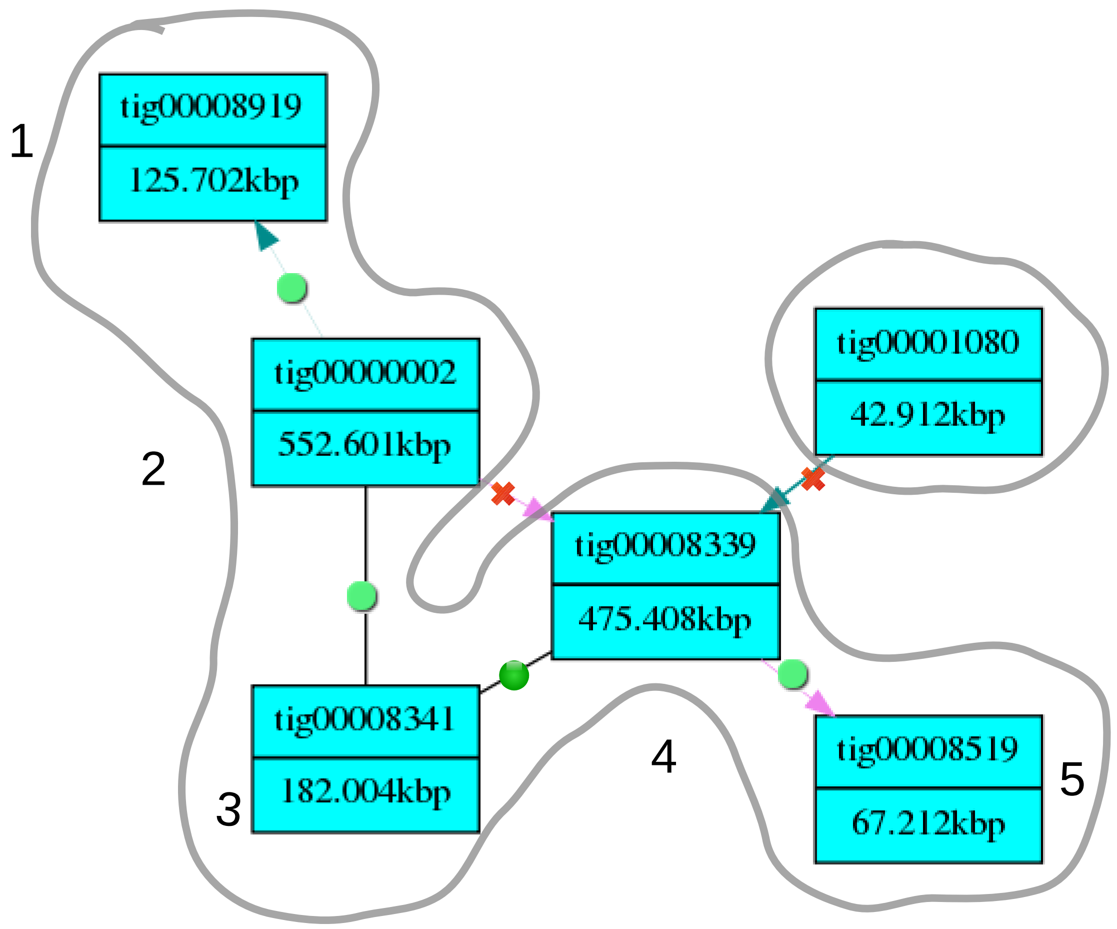

1
tig00008919
125.702kbp
2
tig00000002
552.601kbp
tig00001080
42.912kbp
tig00008339
475.408kbp
tig00008341
182.004kbp
3
4
tig00008519
67.212kbp
5

b)

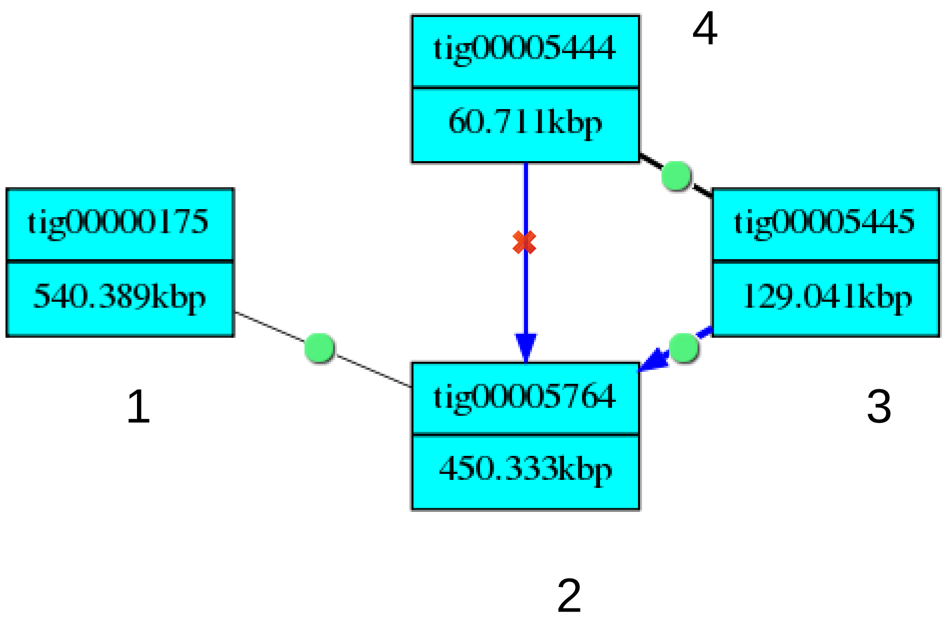

tig00005444
60.711kbp
4
tig00000175
540.389kbp
1
tig00005445
129.041kbp
3
tig00005764
450.333kbp
2

c)

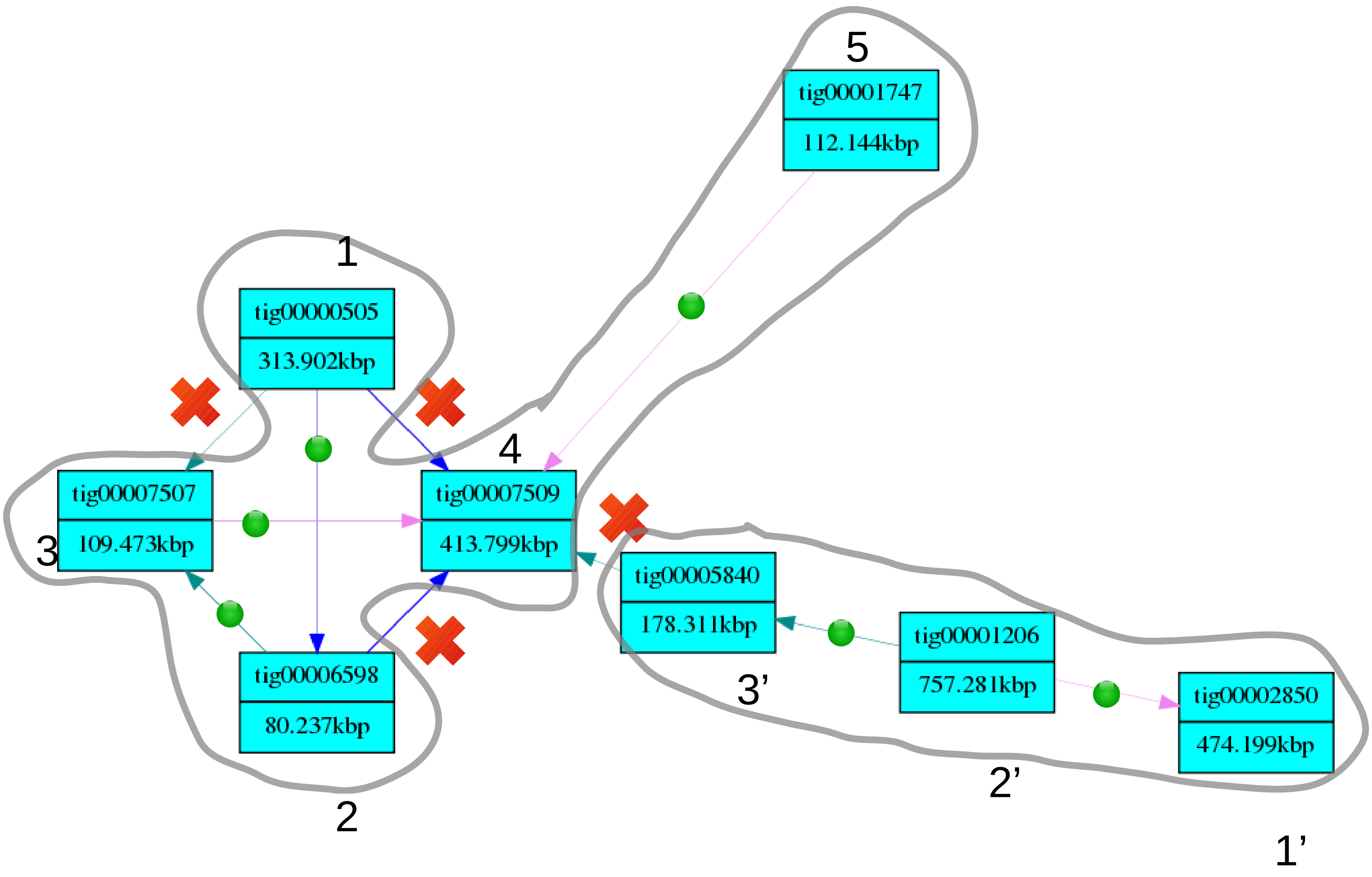

5
tig00001747
112.144kbp
1
tig00000505
313.902kbp
4
tig00007507
109.473kbp
3
tig00007509
413.799kbp
tig00005840
178.311kbp
tig00001206
757.281kbp
tig00002850
474.199kbp
tig00006598
80.237kbp
3'
2
2'
1'

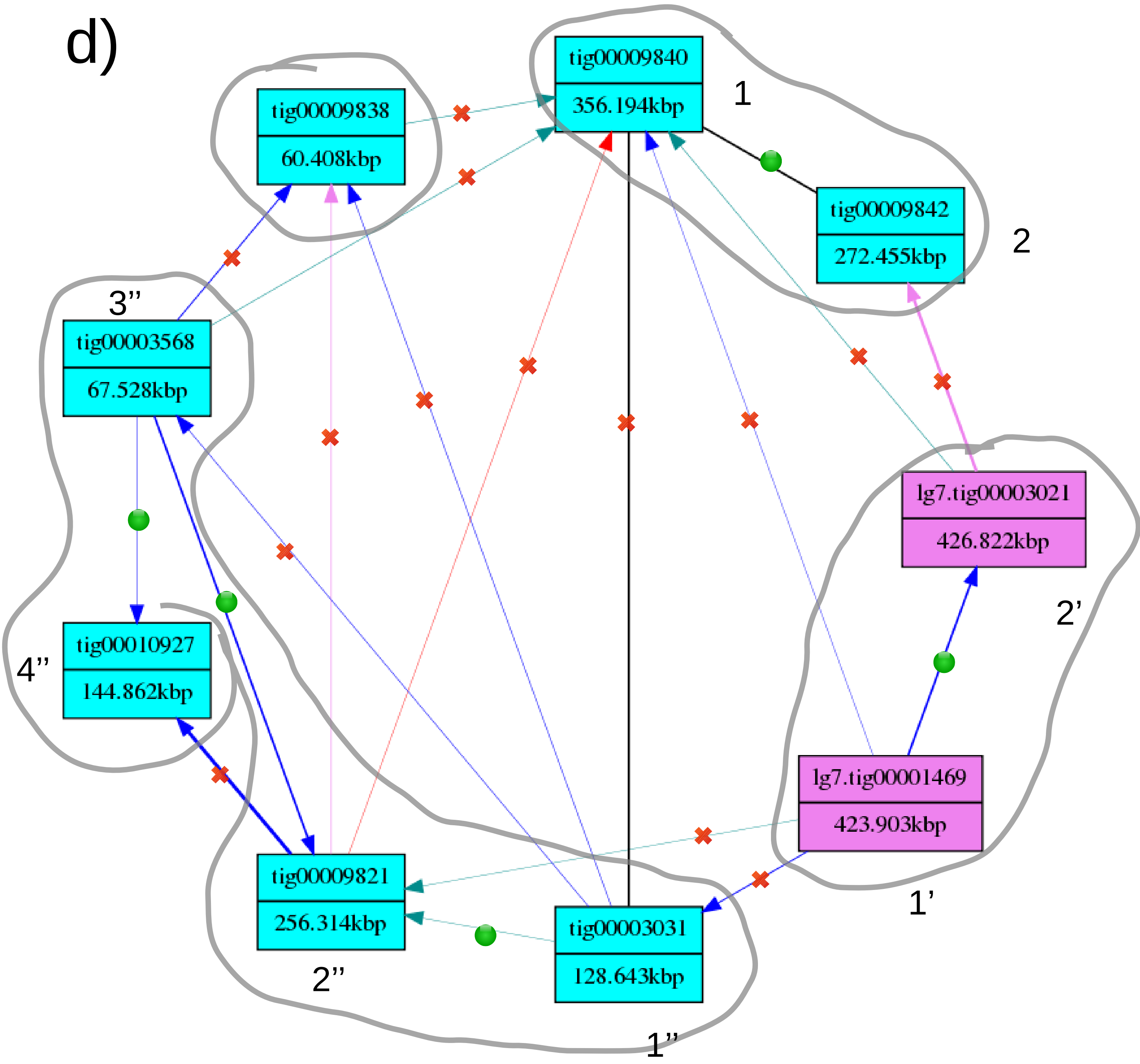
d)
tig00009840
356.194kbp
1
tig00009838
60.408kbp
tig00009842
272.455kbp
2
3''
tig00003568
67.528kbp
lg7.tig00003021
426.822kbp
2'
tig00010927
144.862kbp
4''
lg7.tig00001469
423.903kbp
tig00009821
256.314kbp
tig00003031
128.643kbp
1'
2''
1''

e)

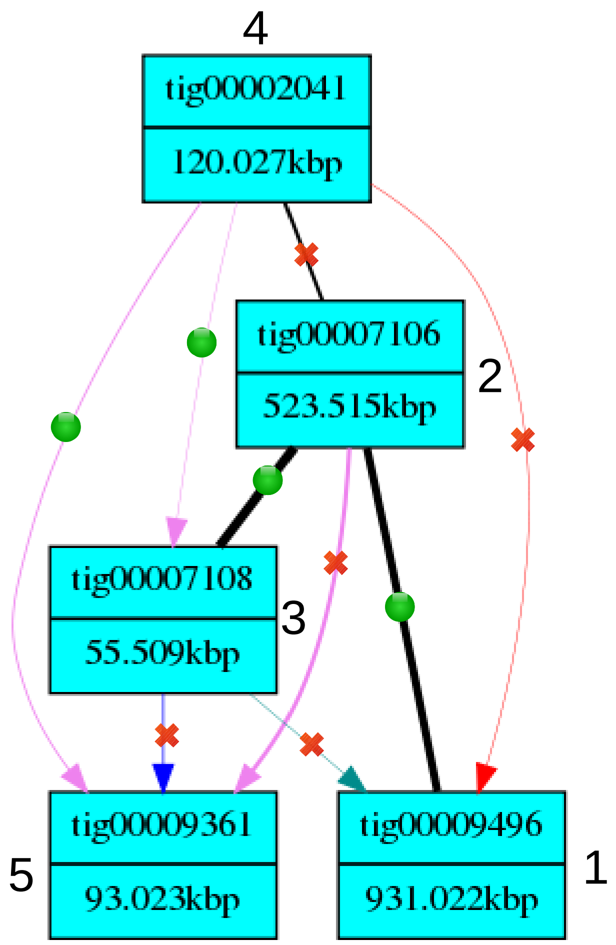
4
tig00002041
120.027kbp
tig00007106
523.515kbp
2
tig00007108
55.509kbp
3
tig00009361
93.023kbp
5
tig00009496
931.022kbp
1

f)

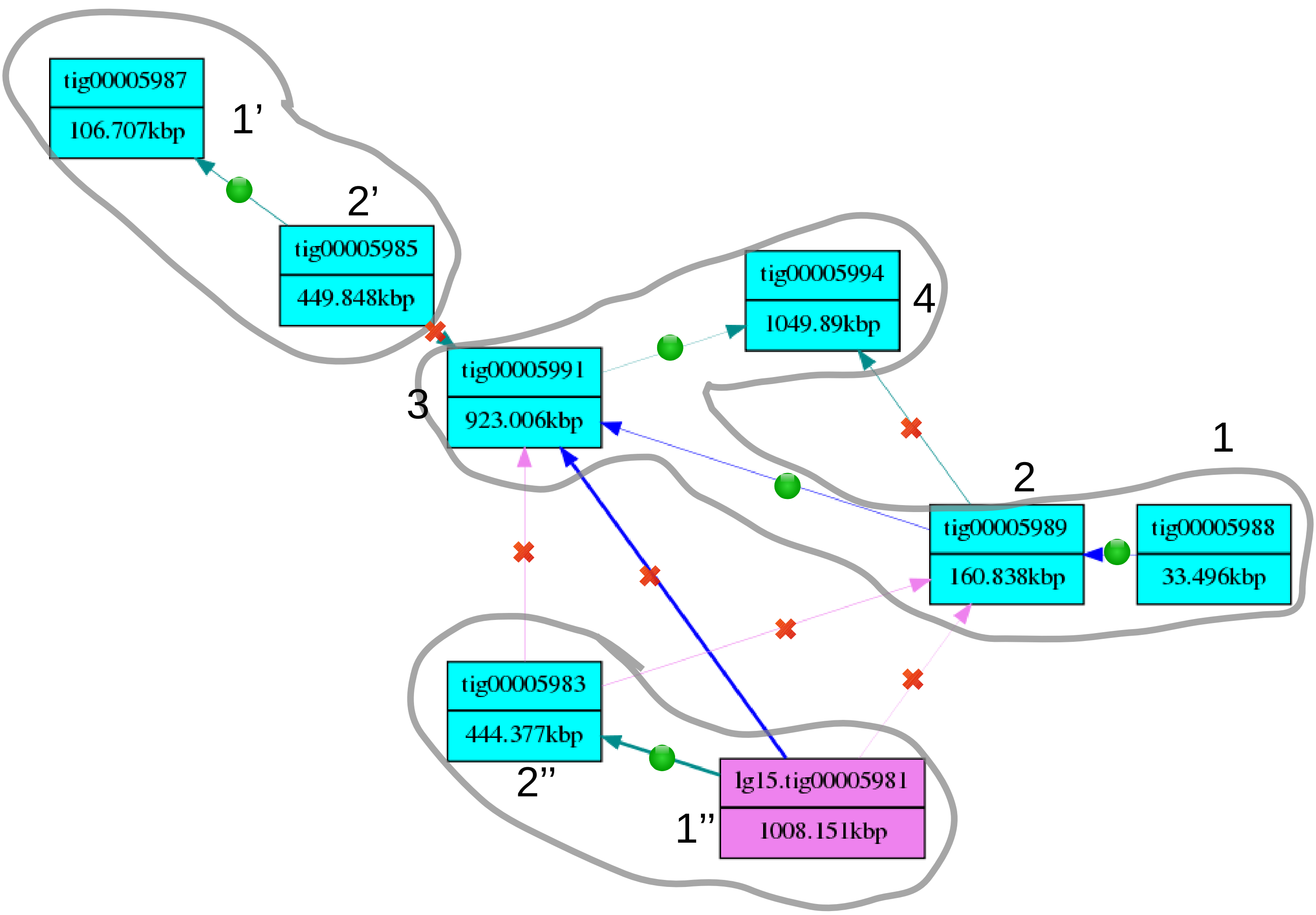

tig00005987
106.707kbp
1'
2'
tig00005985
449.848kbp
tig00005994
1049.89kbp
4
tig00005991
923.006kbp
3
1
2
tig00005989
160.838kbp
tig00005988
33.496kbp
tig00005983
444.377kbp
2''
lg15.tig00005981
1008.151kbp
1''
